# *Bos indicus* cattle resistant to digital dermatitis exhibit a balanced proinflammatory cytokine response upon exposure to *Treponema phagedenis* in contrast to *Bos taurus* cattle

**DOI:** 10.1186/s13567-026-01848-1

**Published:** 2026-10-07

**Authors:** Thomas Démoulins, Thatcha Yimthin, Laura Tassera, Isabelle Brodard, Lukas Eggerschwiler, Andrew Hemphill, Joerg Jores, Peter Kuhnert

**Affiliations:** 1 Institute of Veterinary Bacteriology, Department of Infectious Diseases and Pathobiology, Vetsuisse Faculty, University of Bern, 3012 Bern, Switzerland; 2 Graduate School for Cellular and Biomedical Sciences, University of Bern, Bern, Switzerland; 3 Research Contracts Animals Group, Agroscope, 1725 Posieux, Switzerland; 4 Institute of Parasitology, Department of Infectious Diseases and Pathobiology, Vetsuisse Faculty, University of Bern, 3012 Bern, Switzerland; 5 Multidisciplinary Center for Infectious Diseases (MCID), University of Bern, 3001 Bern, Switzerland

**Keywords:** *Bos indicus*, *Bos taurus*, bovine digital dermatitis, *Treponema phagedenis*, *Dichelobacter nodosus*, proinflammatory immune response, matrix metalloproteinase

## Abstract

Bovine digital dermatitis (BDD) is a globally distributed infectious hoof disease that negatively impacts the wellbeing and productivity of the affected animal. *Treponema* species and in particular *Treponema phagedenis* are the microbes strongly associated with the disease. Recently, it has been reported that *Bos indicus* cattle are more resistant to BDD and to the colonization by *Treponema phagedenis* in contrast to many *Bos taurus* breeds. This study investigated whether *Bos indicus* mount a different immune response to *Treponema phagedenis* compared with *Bos taurus* cattle, to better understand the different disease outcome in the two subspecies. Therefore, we exposed freshly isolated primary bovine blood cells to *Treponema phagedenis* and dissected subsequent bovine immune responses. The comparison between the two subspecies revealed significant different immune responses to *Treponema phagedenis*. We observed a stronger Th1/proinflammatory cytokine induction in *Bos taurus* animals mainly driven by CD4^+^ T and CD21^low^ B cells in secreting IFN-γ. We assume that these distinct initial immune responses are linked to the control of *Treponema phagedenis* and the outcome of BDD. Additionally, we observed significant higher levels in the expression of the matrix metalloproteinase-9 by peripheral blood mononuclear cells in *Bos indicus* cattle, which likely benefits the host response to *Treponema phagedenis*.

**Supplementary Information:**

The online version contains supplementary material available at https://doi.org/10.1186/s13567-026-01848-1.

## Introduction

Cattle are the mammalian species with the most biomass [1]. They are a high value livestock species providing a big share of animal protein for nutrition. Among diseases affecting cattle, bovine digital dermatitis (BDD), also known as Mortellaro disease, is a globally endemic, painful hoof disease, causing active and chronic foot skin lesions, most often found between the heel bulbs, resulting in lameness. Besides compromising animal welfare to a large extend, BDD impacts productivity and leads to reduced milk production, infertility, weight loss, and thereby an increased risk of culling [2–5].

Active BDD lesions usually do not heal well and progress to chronic or recurrent lesions [6]. Application of topical treatment with antibiotics, such as oxytetracycline (OTC), in affected cattle is only partially effective to eliminate the infection [7] and to decrease the ongoing inflammation [8]. The reason why most BDD lesions remain inflamed and progress to chronic conditions despite antibiotic treatment remains unknown. It is now acknowledged that BDD is a polymicrobial disease, and *Treponema* species and predominantly *Treponema phagedenis* are consistently present in lesions [9–12]. Although to a lesser extent *Dichelobacter nodosus* [13, 14] has been isolated from BDD lesions. However, the role of the latter is controversial since it can be regularly isolated from apparently healthy cattle. Therefore, it could be considered a commensal since most *Bos taurus* cattle are colonized with benign strains of *Dichelobacter nodosus* (*aprB2*-positive), while the virulent pathotype (*aprV2*-positive) causing ovine footrot has so far not been reported in Swiss cattle [15–18].

About 8000 and 10 000 years ago, *Bos indicus* and *Bos taurus* cattle have been domesticated in the Indus valley and in the fertile crescent, respectively [14, 19–22]. *Bos indicus* animals have a distinctive morphology with fatty hump on their shoulders, large ears, and dewlap, but they also fostered new and multifaceted adaptive strategies due to their acclimatization to robust hydroclimate variability and drier and less hospitable zones [23]. Certainly, they have higher heat-resistance, can sustain poor nutrition, and show increased resistance to several pathogenic parasites such as babesiosis [24], ticks [25], and nematodes [26] compared with *Bos taurus* cattle.

About 30 years ago, a number of *Bos indicus* cattle were introduced to Switzerland and kept in suckler cow husbandry. We investigated feet from Swiss *Bos indicus* cattle and reported for the first time that they do not display BDD lesions and are significantly less colonized by bacterial hoof microbes such as *Treponema* species and *Dichelobacter nodosus* [17]. This indicates genetic resistance of *Bos indicus* to BDD compared with *Bos taurus*. The present study aimed to test whether the immune response to hoof microbes such as *Treponema phagedenis* and *Dichelobacter nodosus* differs between *Bos indicus* and *Bos taurus* cattle, which is in line with the reported resistance of *Bos indicus* cattle to colonization with BDD pathogens. We used an ex vivo laboratory platform promoting the principles of Replacement, Reduction, and Refinement (3Rs) that already provided meaningful insights into a number of ruminant–microbe interactions [27–30]. We found that immune cells derived from *Bos indicus* animals exhibit a dichotomic response to *Treponema phagedenis*, initially controlling proinflammatory cytokine release while producing elevated levels of matrix metalloproteinases, providing meaningful insight to better understand the resistance to BDD for this specific cattle breed.

## Materials and methods

### Bovine blood collection

We took advantage of the presence of *Bos indicus* (*n* = 8, Holstein Friesian breed, aged 3–5 years) and *Bos taurus* (*n* = 8, Zebu breed, aged 5 months–17 years) cattle in the vicinity of Bern for sampling (Additional file 1) since the infection pressure and livestock production systems are similar and allow a side-by-side analysis. Though the two cohorts were housed in variable farming systems and farming procedures, which might eventually impact some of our findings. Indeed, blood of Holstein Friesian *Bos taurus* cattle was collected at the Agroscope research facility in Posieux, Switzerland, whereas blood of *Bos indicus* cattle was collected at a farm in the canton of Bern. Enrollment of animals in the study was randomized, with no blocking and, to our knowledge, no prior clinical reports of BDD-related disease. We used jugular vein puncture and ethylenediaminetetraacetic acid (EDTA) as well as serum vacutainer tubes from Becton Dickenson for blood collection.

### Serological screening of previous exposure with *Treponema phagedenis*

At the time of this study, enrolled animals had no sign of BDD-related disease. Previous exposure to *Treponema phagedenis* was investigated serologically on all animals using the BioLisa kit Treponema Ab (Biosellal, France). Serum from venous blood to be tested was collected by jugular vein puncture from the animals using standard methods employing vacutainer serum tubes as stated above.

### *Treponema phagedenis* and *Dichelobacter nodosus* propagation and titration

The bovine *Treponema phagedenis* type strain B43.1^T^ isolated from a BDD lesion in Switzerland in 2018 was used in this study [31]. It was cultured in 5 mL of oral treponeme enrichment broth (OTEB; Anaerob Systems) under anaerobic conditions at 37 °C for 2–3 days. Subsequently, 2 × 2 mL of the liquid culture were washed twice with 10 mL of phosphate-buffered saline (PBS) to remove visible and invisible OTEB components. The bacterial pellets were then resuspended in 2 × 2 mL of 0.85% NaCl, followed by a determination of the cell density which was about McFarland 1. After another centrifugation step at 4000 × *g* for 5 min, the supernatant was discarded and the bacterial pellets were carefully resuspended and pooled in 200 µL Dulbecco’s modified Eagle’s medium (DMEM; Life Technologies). This yields a starting solution of approximately 10^9^ cells/mL as determined by quantitative real-time polymerase chain reaction (RT-PCR) [32].

The two ovine *Dichelobacter nodosus* field strains DN4.5RT1 (benign for sheep) and DNT71KR (virulent for sheep) were cultured on Fastidious Anaerob Agar (FAA; Neogen) plates supplemented with 5% sheep blood and 5% rabbit serum under anaerobic conditions at 37 °C for 2–3 days. The bacteria were resuspended in 3 mL of 0.85% NaCl solution until a density of McFarland 4 was reached. Cells were pelleted by centrifugation at 4000 × *g* for 5 min, the supernatant was discarded, and the cell pellet was resuspended in 10 mL of PBS. Cells were again pelleted by centrifugation at 4000 × *g* for 5 min, the supernatant was discarded, and the cell pellet was finally carefully resuspended in 1 mL of DMEM to yield a stock solution of approximately 10^9^ cells/mL as determined by quantitative RT-PCR [33]. Where not indicated otherwise, the benign strain was used in experiments.

For quantification of live bacterial cells propidium monoazid (PMA) was applied prior to the RT-PCR. PMA enters bacteria with a compromised cell wall and intercalates with DNA that then impairs RT-PCR, which eventually does not detect dead bacteria. PMA has no effect on intact viable bacteria, allowing RT-PCR detection and quantification of live microorganisms. Thus, 10 µL of supernatants from stimulated PBMCs were collected 16 h post infection (pi) and transferred to a 1.5 mL reaction tube and mixed with 40 µL of 0.85% NaCl. In a darkened room, 1 µL of PMAxx™ Dye (Biotium; 20 mM in H_2_O) diluted 1:4 with pyrogen-free H_2_O was added to the sample and then vortexed. This was followed by a 3-min incubation in the dark. All samples were then exposed to LED light (white light, 100 W, 6000 K, 8500 lm) for 5 min at a distance of 20 cm on ice. After adding 450 µL of spin or vacuum (SV) buffer (4 M guanidine thiocyanate and 0.01 M Tris–HCl pH 8.0), the samples were stored overnight at 4 °C. DNA extraction and RT-PCR were performed as described previously [32, 33].

### Isolation of bovine peripheral blood mononuclear cells (PBMCs)

30–50 mL of EDTA blood was used for PBMC isolation. Blood was first centrifuged at 1000 × *g* for 20 min. Then the buffy coat was collected and diluted with PBS containing 1 mM UltraPure^™^ EDTA (Invitrogen) to a ratio of 1:1 before being layered onto Ficoll-Paque (1.077 g/mL; GE Healthcare Europe GmbH). After centrifugation at 800 × *g* for 25 min, PBMCs were collected and washed twice with cold PBS containing 1 mM EDTA (Invitrogen) employing centrifugation steps at 350 × *g* for 10 min. If needed, erythrocytes were lysed by resuspending the pellet with 1–2 mL of H_2_O and washed immediately with 48 mL of cold PBS containing 1 mM EDTA and centrifuged at 350 × *g* for 10 min. The final washing step was done at 250 × *g* for 10 min to remove platelets.

### Infection of bovine PBMCs with *Treponema phagedenis* or *Dichelobacter nodosus*

PBMC infection employed flat-bottom six-well plates (TPP, Switzerland). Per well, 5 × 10^6^ PBMCs were cultured in 3 mL DMEM supplemented with 10% fetal bovine serum (FBS) (Life Technologies). Importantly, all tests were run at 38.5 °C (bovine body temperature) with 5% CO_2_.

Infection with *Treponema phagedenis* or *Dichelobacter nodosus* was done at a high multiplicity of infection (MOI) = 1 or a low MOI of 0.1 for 20 h, based on previous study [28] and preliminary experiments.

At 0 h and 16 h pi, 50–100 μL of cell culture supernatant was collected and frozen at −20 °C for subsequent cytokine and chemokine secretion measurement using a bead-based multiplex immunoassay (see below). At 16 h pi, 10 μg/mL of Brefeldin A (ThermoFisher) was added to the medium to block cytokine secretion and incubation was extended for another 4 h, to allow the de novo cytokine synthesis measurement employing flow cytometry (FCM) intracellular staining.

### Multiparameter flow cytometry (FCM) assay

Application of multiparameter FCM assay has been described previously [28], apart from very few changes of antibodies and fluorophores: in-house conjugation of CD3 employed Zenon Alexa Fluor 568 labeling kit (instead of the Alexa Fluor 532 Antibody Labeling Kit), and CD45RO (clone IL-A116) replaced CD44 (Additional file 2). Otherwise, combination staining and gating strategies remained unchanged for analyzing monocytes (classical, intermediate, and nonclassical), conventional dendritic cell [cDCs; Type 1 cDCs [cDC1s] and Type 2 cDCs (cDC2s)], pDCs, γδ T cells, NK cells, CD4^+^ and CD8^+^ T cells, and B cells (Additional file 3). For the acquisitions, cells from the whole samples were accumulated. For the fold-change analysis of activation/maturation marker following infection, the mean fluorescence intensity (MFI) measured in stimulated samples for a given animal was normalized to the MFI measured in an unstimulated sample from that same animal.

FCM acquisitions were performed on a Cytek Aurora (Cytek Biosciences) using the SpectroFlo software with autofluorescence extraction, spectral unmixing with single-stained reference control for every fluorophore used in the panels, plus an unstained control, and further analyzed with FlowJo 10.9.0 (TreeStar).

### Multiplex bead-based immunoassay

We quantified secreted cytokines and chemokines in the supernatants using the commercial MILLIPLEX Bovine Cytokine/Chemokine Magnetic Bead Panel 1—Immunology Multiplex (Merck) according to suppliers’ recommendations as reported elsewhere [28]. Of note, chemokines are named in the figures with their “historical names”, which adhere to the names used in the manufacturer’s manual. However, the recent nomenclature states that the new names for the chemokines are CCL3 (C–C motif ligand 3), CXCL10 (C–X–C motif ligand 10), CCL2, and CCL4 for MIP-1α, IP-10, MCP-1, and MIP-1β, respectively.

### Measurement of bovine matrix metalloproteinase 9

Concentrations of bovine matrix metalloproteinase-9 (MMP-9) in the supernatants of PBMCs were determined using a Bovine MMP-9 enzyme-linked immunosorbent assay (ELISA) kit (AssayGenie) and Tecan Spark plate reader according to vendors’ instructions. Additionally, we re-analyzed *Bos indicus* and *Bos taurus* RNA sequencing (RNA-Seq) data published recently [29] with respect to the transcriptional levels of the bovine MMP-9.

### Statistical analysis and reproducibility

For all experiments, PBMCs were isolated from blood of eight animals to study their response to *Treponema phagedenis* and *Dichelobacter nodosus*, and individual data points are displayed. Statistical analysis was done using the GraphPad Prism 8 software (GraphPad software, La Jolla, CA, USA). To determine differences between groups, two-tailed Mann–Whitney *U* tests, nonparametric paired Wilcoxon tests, or repeated-measures one-way analyses of variance (ANOVAs) followed by Tukey’s post hoc test were used, as appropriate. Associations were tested using the Pearson rank correlation test. A *p* < 0.05 was considered statistically significant.

## Results

### Previous exposure to *Treponema phagedenis*

At the time of the study, the cattle (*n*_*Bos taurus*_ = 8; *n*_*Bos indicus*_ = 8) enrolled in the study were apparently healthy, were randomly selected, and bled and later tested for their seroprevalence toward *Treponema phagedenis*. In the absence of a commercial serological test for *Dichelobacter nodosus*, we were not able to test seroprevalence for the latter microbe. Serological analysis confirmed the presence of *Treponema*-specific antibodies in two *Bos taurus* and one *Bos indicus*, indicating previous exposure to this pathogen in the three seropositive animals (Additional file 1). These frequencies were in line with published data on their local prevalence [17].

### Intrinsic capacity of peripheral blood mononuclear cells from *Bos taurus* or *Bos indicus* animals to respond to immune stimulators

**Figure 1:**
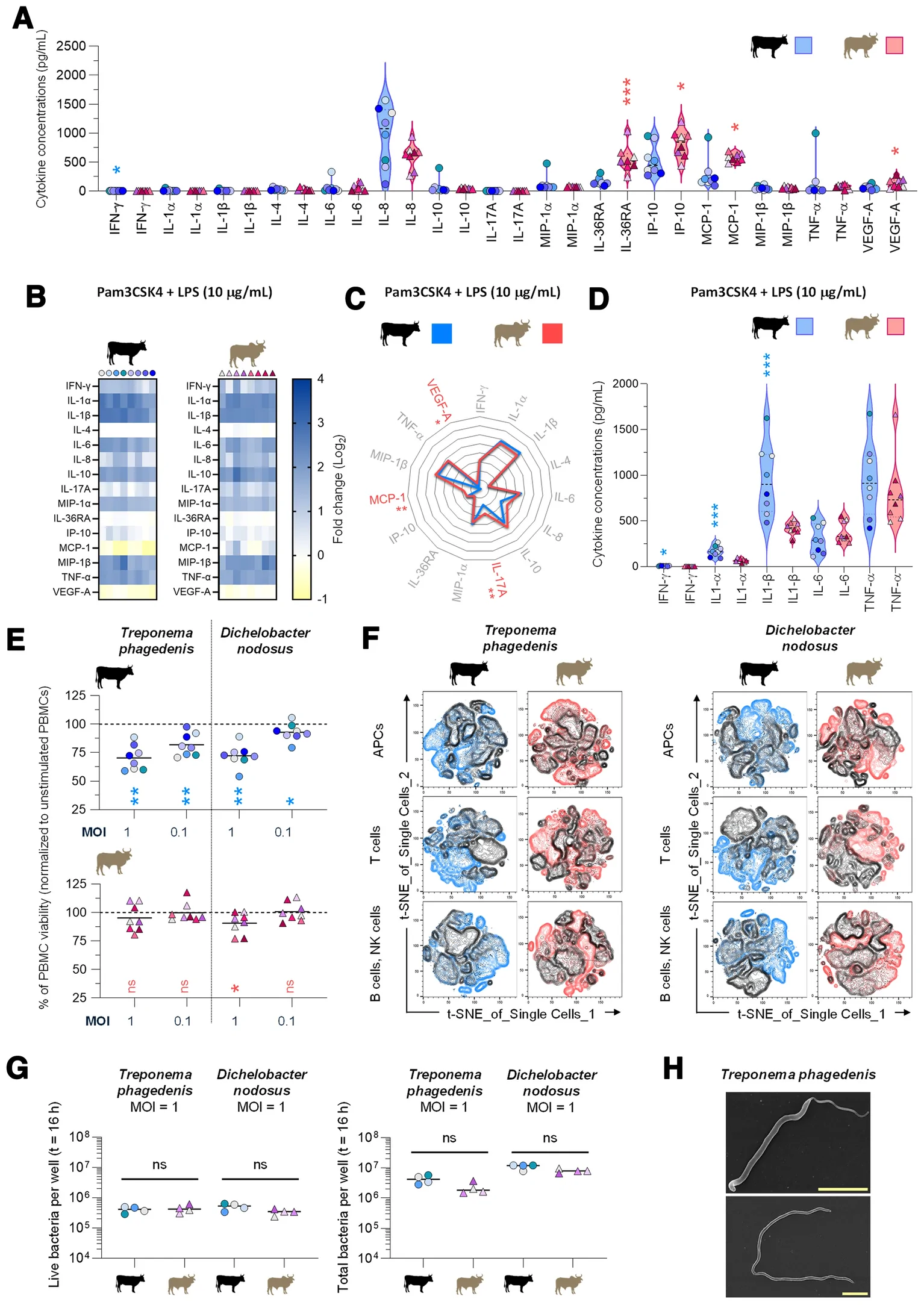
**Intrinsic capacity of peripheral blood mononuclear cells from** ***Bos taurus***or ***Bos indicus*** **animals to respond to immune stimulators**. **A** Cytokine concentrations (pg/mL) in the serum of bled *Bos taurus* (*n* = 8) and *Bos indicus* (*n* = 8) animals. **B**–**D** Cytokine/chemokine secretion in supernatants of PBMC cultures, using multiplex immunoassay. PBMCs from eight individual animals were either let for 16 h unstimulated (reference points for the assay) or stimulated with “Pam3CSK4 + LPS” (“bacterial infection”). **B** A single measurement was done per sample tested, and each symbol represents an individual animal. Heat map shows log_2_-fold changes in concentration of 15 cytokines/chemokines. For a given cytokine/chemokine, normalization was as follows: [concentration for a given animal]/[average concentration of reference points (∅) for eight animals]. **C** Radar plots showing the mean of eight animals per group of log_2_-fold changes in concentration. **D** The concentrations (pg/mL) of the major proinflammatory cytokines following stimulation of primary blood cells by “Pam3CSK4 + LPS” are presented. The violin plots show the values obtained from the eight animals. **E**–**G** PBMCs from eight individual animals per cattle subspecies were either let for 16 h unstimulated (reference points for the assay), stimulated with *Treponema phagedenis* (MOI = 1 or MOI = 0.1) or *Dichelobacter nodosus* (MOI = 1 or MOI = 0.1). **E** Impact of bacterial infection on viable PBMC frequencies. A primary gate (P1) was set on forward scatter (FSC)-A versus side scatter (SSC)-A, then, the percentage of living PBMCs (negative for live/dead marker) was quantified by FCM. Cells were either unstimulated or stimulated with either *Treponema phagedenis* (MOI = 1 or MOI = 0.1) or *Dichelobacter nodosus* (MOI = 1 or MOI = 0.1). Each symbol represents an individual animal and is the average of three independent measurements (“antigen-presenting cells”, “T cells”, and “B cells, NK cells” panels). Experimental conditions were compared using nonparametric paired Wilcoxon tests. Asterisks indicate significance levels. ^*^p < 0.05; ^**^p < 0.01. *ns* not significant. **F** The live primary blood cells from eight animals were merged to create a single t-distributed stochastic neighbor embedding (t-SNE) map with the signal strength of all phenotypic markers for the three staining combinations: “APCs,” “T cells,” and “B cells, NK cells.” A “time” gate was used to gate out irregularities in sample acquisition. Then, a primary gate (P1) was set on FSC-A versus SSC-A, after which the dead cell population (positive for live/dead) was excluded, followed by a FSC-H/FSC-A contour plot (to exclude doublets from the analysis). Blue islands show PBMC phenotypic change from infected samples derived from *Bos taurus* animals, whereas red islands indicate PBMC phenotypical change in cells derived from *Bos indicus* animals. The t-SNE algorithm used perplexity value of 30 and 1000 iterations. Black zones represent unstimulated cells. **G** Number of live (left side) and total (right side) bacteria per well in supernatant from infected primary blood cells. PBMCs from four individual animals per group were stimulated for 16 h with *Treponema phagedenis* or *Dichelobacter nodosus* (MOI = 1). Supernatants were collected, and the number of bacteria were determined using a RT-PCR assay as described previously [32]. Each symbol represents an individual animal. Statistics employed Mann–Whitney *U* tests. **H** Scanning electron micrographs of *Treponema phagedenis* at different magnifications (20 000× at the top, 10 000× at the bottom). Scale bar = 2 μm. Blue asterisks indicate significant higher levels in *Bos taurus* animals, whereas red asterisks indicate significant higher levels in *Bos indicus* animals. Assays were carried out at bovine body temperature of 38.5 °C.

All animals enrolled in this study were raised in Switzerland, but *Bos taurus* and *Bos indicus* cohorts were housed in different farming systems and procedures, raising the possibility that external factors could affect ex vivo immune responses of the cattle. To evaluate this, we first measured the cytokine concentration in the serum of all animals enrolled in the study. Background cytokine levels were comparable between the two cattle subspecies for 10 out of 15 cytokines. Interestingly, a slightly—but significantly—higher concentration of IFN-γ was measured in the serum of *Bos taurus*, whereas IL-36RA, IP-10, MCP-1, and VEGF-A were found at higher level in the serum of *Bos indicus* animals (Figure 1A). Those background differences support the notion that *Bos indicus* and *Bos taurus* cattle are likely to exhibit distinct ex vivo responses to hoof microbes.

Then, we measured the capacity of peripheral blood mononuclear cells (PBMCs) to respond to synthetic TLR agonists (i.e., Pam3CSK4 and LPS). Again, the induction profiles were quite superimposable between *Bos taurus* and *Bos indicus* animals, except for VEGF-A, IL-17A, and MCP-1 (Figure 1B, heat map showing all individual animals; Figure 1C, radar plots showing the means of eight animals). Of note, when we took into account the concentrations rather than induction (meaning no more consideration of background levels as in Figure 1B and C), then *Bos taurus* individuals clearly showed an enhanced secretion of proinflammatory cytokines (significant despite the high interindividual variability) (Figure 1D). In conclusion, although most of the immune parameters investigated were comparable in both cattle subspecies, we were able to identify slight differences—both at the steady-state level and at the capacity to respond to TLR agonists.

These differences prompted us to test the impact of bacteria exposure on PBMC viability. In a pilot experiment, we assessed the responsiveness of *Bos taurus* derived antigen-presenting cells (APCs) to both *Treponema phagedenis* and *Dichelobacter nodosus* to determine optimal MOIs [MOI = 1 (“high”), MOI = 0.1 (“low”)] and time points (*t* = 20 h). Then, for *Bos taurus* animals, significant loss of cell viability was seen for both *Treponema phagedenis* and *Dichelobacter nodosus*, regardless of the MOI. This differed to what was observed for *Bos indicus* animals, where cell viability decreased only following exposure to *Dichelobacter nodosus* at high MOI (Figure 1E). The next step was to ensure that PBMCs derived from both cattle subspecies were activable in response to those two live hoof microbes. This was first investigated with the t-distributed stochastic neighbor embedding (t-SNE) algorithm [visualization of high dimensional data in a two-dimensional (2D representation]. Clearly, blue and red islands (for *Bos taurus* and *Bos indicus*, respectively), were seen in all stimulated samples, regardless antibody combination staining (“APCs,” “T cells” or “B cells, NK cells”) or bacterial agent (*Treponema phagedenis* or *Dichelobacter nodosus)*, signifying high phenotypic changes (Figure 1F). Furthermore, *Treponema phagedenis* and *Dichelobacter nodosus* did not show a clear difference in their numbers after having interacted with PBMCs derived from either *Bos taurus* or *Bos indicus* individuals, indicative of equivalent capacity of their PBMCs to control the tested hoof microbes (Figure 1G). A scanning electron microscope (SEM) picture of *Treponema phagedenis* is provided in Figure 1H, depicting the pathogen that has a periplasmic flagellum known to activate innate immune system via TLR-2 [34].

### Cytokine induction of peripheral blood mononuclear cells after exposure to *Treponema phagedenis* and *Dichelobacter nodosus*

**Figure 2:**
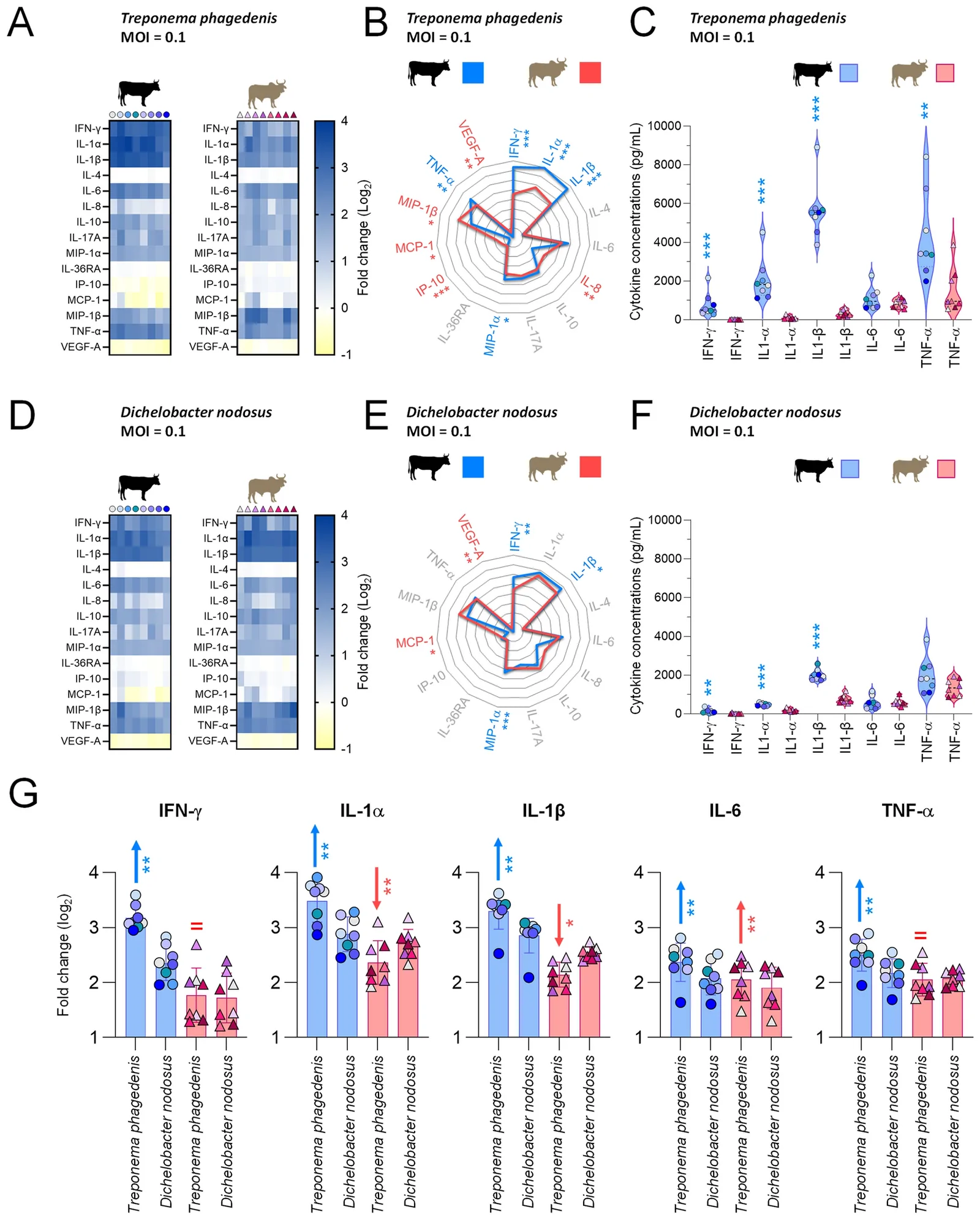
**Cytokine induction of peripheral blood mononuclear cells after infection with *Treponema phagedenis*** **and** ***Dichelobacter nodosus***. PBMCs from eight individual animals per group were either unstimulated or stimulated with *Treponema phagedenis* or *Dichelobacter nodosus* for 16 h. **A**–**C** Interbreed comparison for *Treponema phagedenis* infections were run at ruminant body temperature (38.5 °C). A single measurement was done per sample tested, and each symbol represents an individual animal. **A** Heat map shows log_2_-fold changes in concentration of 15 cytokines/chemokines. For a given cytokine/chemokine, normalization was as follows: [concentration for a given animal]/[average concentration of reference points]. **B** Radar plots showing the mean of eight animals of log_2_-fold changes in concentration. **C** The concentrations (pg/mL) of the major proinflammatory cytokines following primary blood cells infected with *Treponema phagedenis* are presented. The violin plots show the values obtained from the eight animals. **D**–**F** As in **A**–**C** but displaying interbreed comparison for *Dichelobacter nodosus*. **G** Bacterial comparison for *Bos taurus* (blue histograms) and *Bos indicus* (red histograms) animals. An arrow indicates significant differences of pro-Th1 or proinflammatory cytokine induction following *Dichelobacter nodosus* and *Treponema phagedenis* exposure for a given cattle subspecies, whereas no significant influence is indicated by “ = ”. Experimental conditions were compared using Mann–Whitney *U* tests (**B**, **C**, **E**, **F**) or nonparametric paired Wilcoxon tests (**G**). Asterisks indicate significance levels. ^*^p < 0.05; ^**^p < 0.01; ^***^p < 0.001. Blue asterisks indicate significant higher levels in *Bos taurus* animals, whereas red asterisks indicate significant higher levels in *Bos indicus* animals.

Supernatants from the ex vivo infection experiment were collected and investigated for cytokines and chemokines secreted after infection. Upon stimulation with *Treponema phagedenis*, the most noticeable result consisted of detectable levels of a set of pro-Th1/proinflammatory cytokines, most notably IFN-γ, IL-1α, IL-1β, IL-6, MIP-1α (= CCL3), MIP-1β (= CCL4), and TNF-α. However, the inflammatory response observed in *Bos taurus* animals was strongly further enhanced compared with *Bos indicus* animals (Figure 2A, heat map showing normalized value for all individual cattle—clearly darker profile in *Bos taurus* for IFN-γ, IL-1α and IL-1β; Figure 2B, radar plots showing normalized values of the mean of eight animals—clearly increased magnitude for *Bos taurus* for IFN-γ, IL-1α and IL-1β; Figure 2C, histogram showing calculated concentrations of secreted cytokines of all animals).

Results obtained with the interbreed comparison of the ex vivo response to *Dichelobacter nodosus* proved to be relatively minor compared with those obtained with *Treponema phagedenis*. Nevertheless, the interbreed comparison showed some cytokines significantly enhanced in *Bos taurus* animals (i.e., IFN-γ, IL-1β, MIP-1α), while some others were more pronounced in *Bos indicus* animals (i.e., MCP-1, MIP-1β) (Figure 2D, heat map showing all individual cattle; Figure 2E, radar plots showing the mean of eight animals). Similarly to what was observed following blood cell exposure to synthetic TLR ligands (Figure 1D), the magnitude of proinflammatory cytokines IFN-γ, IL-1α and IL-1β was found significantly higher for *Bos taurus* when we considered strictly the concentrations rather than the fold change (Figure 2F), proving once more that, unlike *Bos indicus* animals, they tend to respond in a strong manner to live pathogens. Of note, Additional file 4A–C shows results with PBMC stimulation employing virulent *Dichelobacter nodosus* (DNT71KR isolate): the pattern and magnitude of secreted cytokines/chemokines was comparable to the one raised in response to benign *Dichelobacter nodosus*.

Finally, when the impact of the two distinct hoof microbes on cytokine secretion was compared for a given cattle subspecies, a totally divergent influence was noted: in cells derived from *Bos taurus* animals, the proinflammatory response was strongly enhanced upon *Treponema phagedenis* stimulation (IFN-γ, IL-1α, IL-1β, IL-6, and TNF-α); in PBMCs derived from *Bos indicus* animals, the response upon *Treponema phagedenis* stimulation was either of the same intensity to that quantified with *Dichelobacter nodosus* (IFN-γ, and TNF-α), significantly reduced (IL-1α and IL-1β), or eventually increased (IL-6) (Figure 2G). Clearly, these results reinforce the hypothesis that “overactive inflammation” response to bacteria is a trademark of *Bos taurus* individuals, as reported in BDD active lesions, and they underscore the relevance of breed-specific immunological studies in understanding disease susceptibility and outcome.

### Response of antigen-presenting cells to *Treponema phagedenis* and *Dichelobacter nodosus*

**Figure 3:**
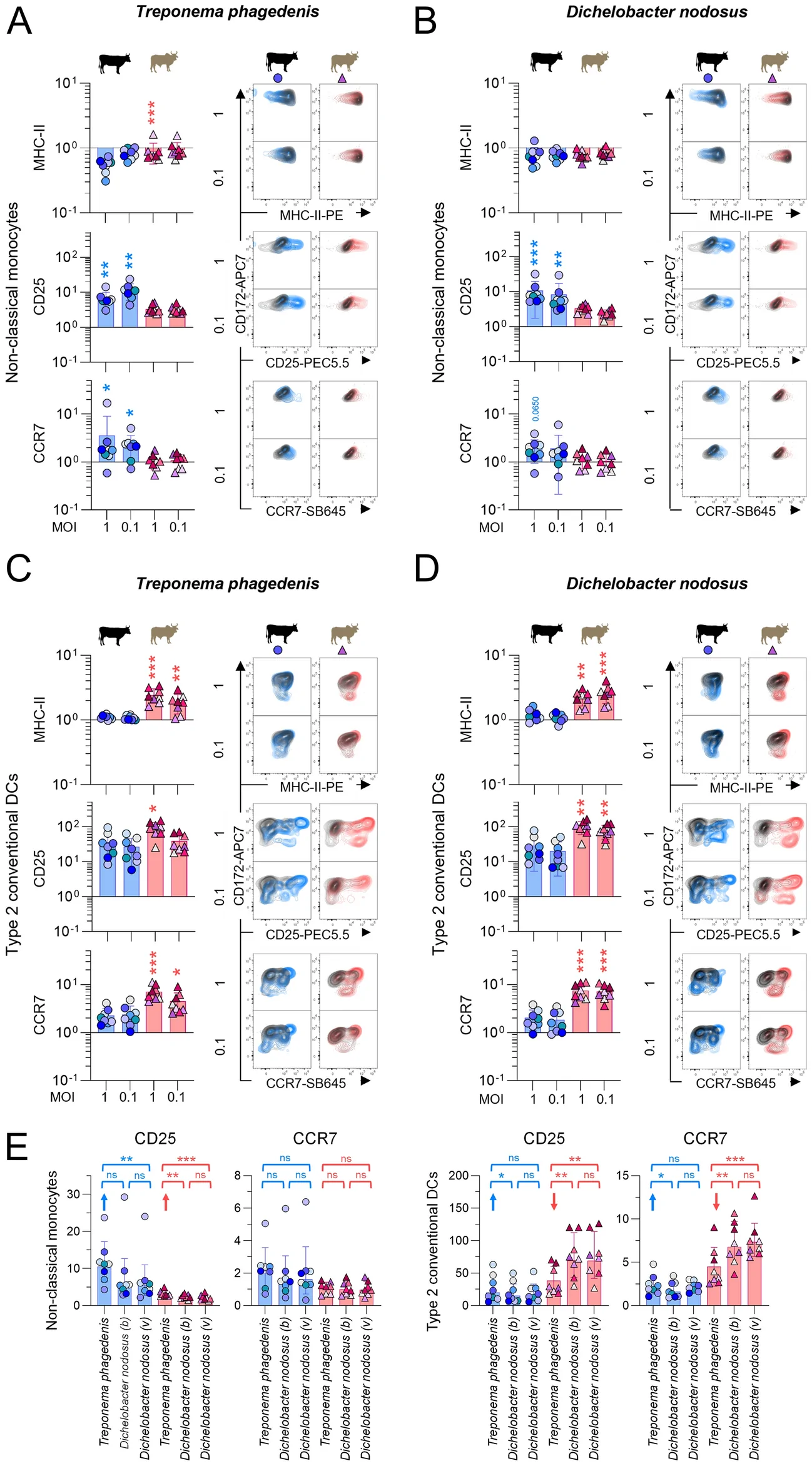
**Response of antigen-presenting cells to *Treponema phagedenis*** **and** ***Dichelobacter nodosus***. PBMCs from eight individual animals per group, whether *Bos taurus* or *Bos indicus*, were either let for 20 h unstimulated (reference points for the assay) or stimulated with *Treponema phagedenis* or *Dichelobacter nodosus*. Data shown for nonclassical monocytes (**A**, **B**) and cDC2s (**C**, **D**). Three activation/maturation markers were considered: MHC-II, related to antigen presentation; CD25, related to cellular activation; and CCR7, related to migration toward draining lymph nodes. For each panel, on the left side, graphs display all individual animals. The fold changes analysis of activation/maturation markers was determined by FCM with FlowJo 10.9.0. Cells from the individual *Bos taurus* and *Bos indicus* animals are represented by separate symbols; for stimulated samples, mean fluorescence intensity (MFI) values are normalized to that obtained with the reference point from the same animal. On the right side are provided representative contour plots overlaying the signal obtained in unstimulated blood cells (black) with the signal obtained in cells exposed to bacteria (blue for activated cells derived from *Bos taurus* animals and red for activated cells derived from *Bos indicus* animals). **E** Bacterial comparison for *Bos taurus* (blue histograms) and *Bos indicus* (red histograms) animals. Arrows indicate significant differences of activation-marker induction following *Treponema phagedenis* compared with benign (b) or virulent (v) *Dichelobacter nodosus *exposure for a given cattle subspecies. Results are displayed for nonclassical monocytes (left panel) and cDC2s (right panel). Experimental conditions were compared using Mann–Whitney *U* tests (**A**–**D**) or repeated-measures one-way ANOVA followed by Tukey’s post hoc test (**E**). Asterisks indicate significance levels. ^*^p < 0.05; ^**^p < 0.01; ^***^p < 0.001. Blue asterisks indicate significant higher levels in *Bos taurus* animals, whereas red asterisks indicate significant higher levels in *Bos indicus* animals. Assays were carried out at bovine body temperature of 38.5 °C.

Overall, the responses of *Bos taurus*- versus *Bos indicus*-derived antigen-presenting cells (APCs) differed significantly in their reactions to both hoof microbes. Nonclassical monocytes derived from *Bos taurus* exhibited MHC-II downregulation and strong and moderate upregulation for CD25 and CCR7, respectively. Nonclassical monocytes derived from *Bos indicus* showed a slight upregulation of CD25 but did not show any impact on MHC-II and CCR7 expression (Figure 3A). Upon *Dichelobacter nodosus* stimulation, nonclassical monocytes from *Bos taurus* showed a clear CD25 upregulation—but this time no consistent modulation of MHC-II and CCR7 markers—whereas nonclassical monocytes from *Bos indicus* only displayed a moderate increase in CD25 expression and did not show activation for the two cell surface markers MHC-II and CCR7 (Figure 3B). Overall, these results indicate that nonclassical monocytes from both cattle subspecies displayed a strong but clearly divergent activation, with *Bos taurus* animals being more responsive to bacterial hoof microbes, particularly to *Treponema phagedenis*.

We then kept our attention on Type 2 conventional dendritic cells (cDC2s). For *Bos taurus* animals, except for the CD25 marker, we failed to detect any clear modulation for activation/maturation markers, regardless of the hoof microbe tested (Figure 3C, D). This time, the results obtained for *Bos indicus* specimens proved to be markedly robust compared with those observed for *Bos taurus* individuals: whatever the hoof pathogen used for stimulation, this led to a stronger CD25 upregulation; to a lesser extent, this observation was also found for MHC-II and CCR7. For clarity, data displayed in Figure 3 focus on nonclassical monocytes and Type 2 conventional dendritic cells (DCs)—two subsets found to be of particular interest in previous studies—whereas the full panel (classical, intermediate and nonclassical monocytes, type 1 and type 2 conventional DCs, and plasmacytoid DCs) is available in Additional files 5 and 6. Taken together, these results related to APCs strongly suggest that the two cattle subspecies are prone to initially combat hoof pathogen infection with qualitatively and quantitatively distinct pathogen recognition and innate immune responses.

Finally, when the impact of distinct hoof microbes on CD25 and CCR7 was compared for a given cattle subspecies, a divergent influence was noted. In *Bos taurus* animals, APCs were more activated following exposure to *Treponema phagedenis* as shown by CD25 upregulation in nonclassical monocytes. In *Bos indicus* animals, APCs were more activated following exposure to *Dichelobacter nodosus*, as shown by CD25 expression levels in cDC2s (Figure 3E).

### The magnitude of proinflammatory response against *Treponema phagedenis* and *Dichelobacter nodosus* negatively correlates with antigen-presenting cell activation

**Figure 4:**
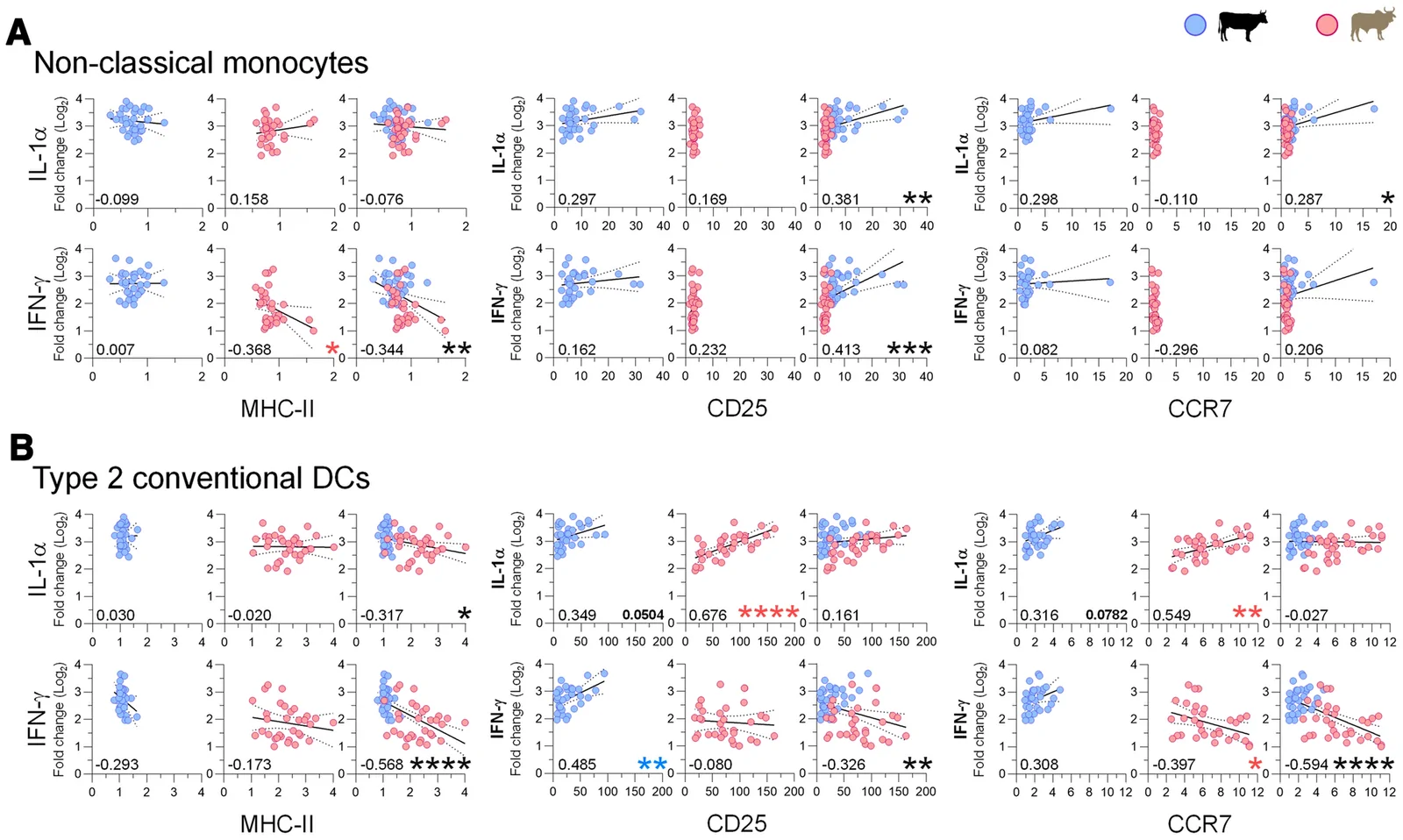
**The level of proinflammatory response (secreted cytokines) against *Treponema phagedenis*** **and *Dichelobacter nodosus*** **negatively correlates with antigen-presenting cell activation (induction of activation/maturation markers)**. **A** Correlation coefficients obtained with secreted cytokines (IL-1α or IFN-γ) calculated as a function of induction of maturation markers (MHC-II, CD25, or CCR7) at the surface of nonclassical monocytes. **B** Correlation coefficient (*r*) obtained with secreted cytokines (IL-1α or IFN-γ) calculated as a function of induction of maturation markers (MHC-II, CD25, or CCR7) at the surface of cDC2s. All correlations are organized as follows: on the left are shown the 32 plots in blue, obtained for *Bos taurus* animals (eight individuals, four experimental conditions: *Treponema phagedenis* MOI = 1 and 0.1; *Dichelobacter nodosus* MOI = 1 and 0.1); in the middle are shown the 32 plots in red, obtained for *Bos indicus* animals (eight individuals, four experimental conditions: *Treponema phagedenis* MOI = 1 and 0.1; *Dichelobacter nodosus* MOI = 1 and 0.1); on the right, the computed plots obtained for all animals (*n* = 16, 64 plots) are displayed. Correlations were tested using the Pearson rank correlation test. Asterisks indicate significance levels. ^*^p < 0.05; ^**^p < 0.01; ^***^p < 0.001; ^****^p < 0.0001. ns, not significant. Blue asterisks indicate significant higher levels in *Bos taurus* animals, whereas red asterisks indicate significant higher levels in *Bos indicus* animals. Assays were carried out at bovine body temperature of 38.5 °C.

To test any significant associations between cytokine secretions and APC activation levels, we combined results obtained with multiplex immunoassay (Figure 2) and multiparameter FCM (Figure 3). This analysis emphasized IFN-γ and IL-1α, two of the highly secreted cytokines following exposure to bacteria, notably showing clear difference of induction depending on *Treponema phagedenis* or *Dichelobacter nodosus* (Figure 2). First, in most of the correlations investigated, data related to *Bos taurus* animals (Figure 4, blue spots) clustered away from data related to *Bos indicus* animals (Figure 4, red spots), confirming that the ex vivo response to microbial agents strongly differs between the two cattle subspecies. Then, when all the plots derived from *Bos taurus* and *Bos indicus* were overlayed, IL-1α induction moderately correlated with activation signs detected in both nonclassical monocytes (Figure 4A) and cDC2s (Figure 4B). Remarkably, all the correlations obtained with IL-1α proved to be moderate compared with what obtained for IFN-γ. Indeed, induction of the latter led to a strong and negative association with MHC-II and CCR7 expression—the best negative correlation being obtained for cDC2s—and a significant correlation with CD25 (positive for nonclassical monocytes, negative for cDC2s). In conclusion, the high magnitude of pro-Th1/proinflammatory response detected in immune cells from *Bos taurus* animals is tightly associated with impaired capacity of APCs to maturate in response to hoof microbes.

### Response of T and B lymphocytes to *Treponema phagedenis* and *Dichelobacter nodosus*

**Figure 5:**
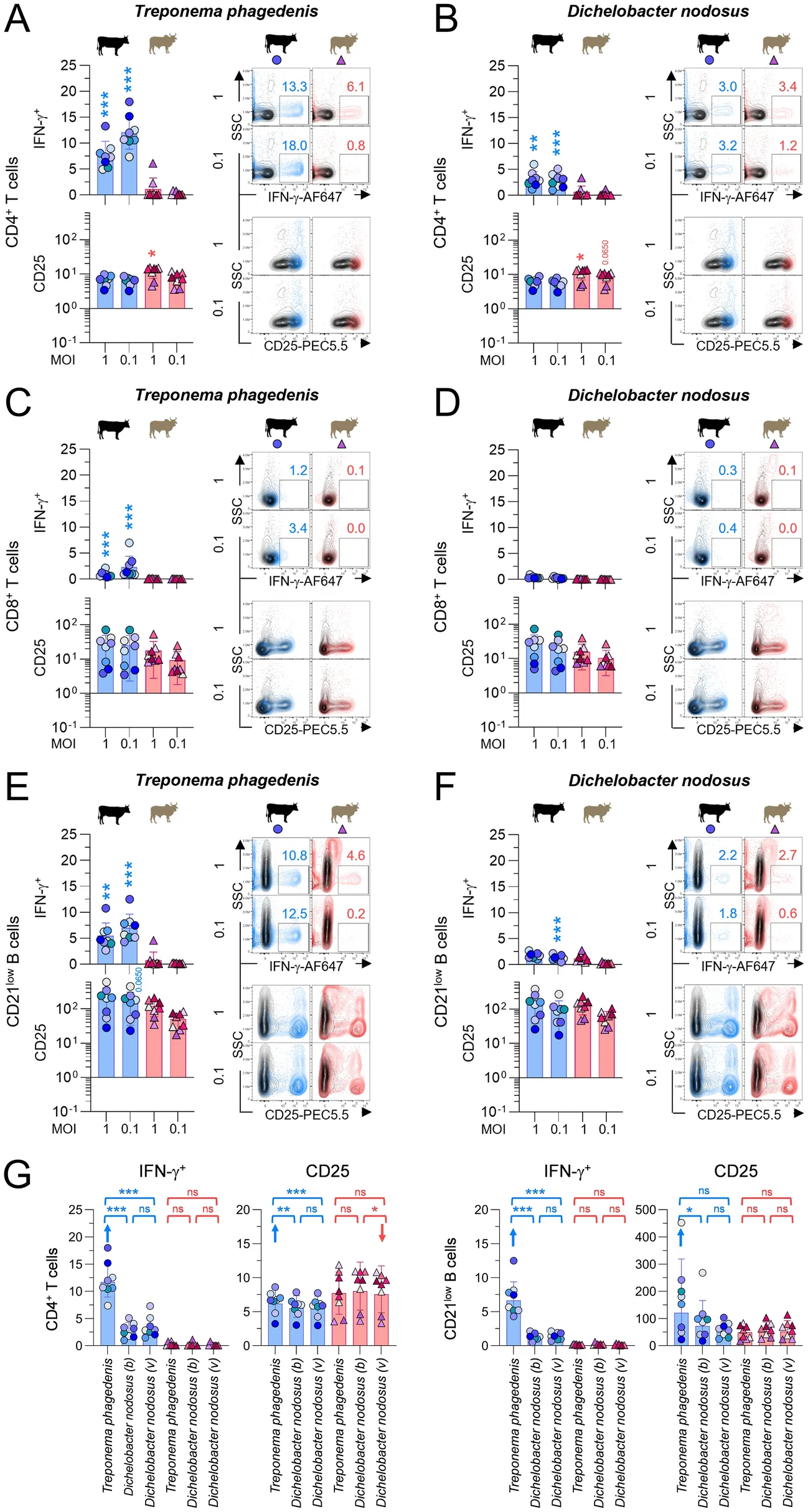
**Response of T and B lymphocytes to *Treponema phagedenis*** **and *Dichelobacter nodosus***. PBMCs from eight individual animals per group, whether *Bos taurus* or *Bos indicus*, were either let for 20 h unstimulated (reference points for the assay) or stimulated with *Treponema phagedenis* or *Dichelobacter nodosus*. Data obtained for CD4^+^ T cells (**A**, **B**), CD8^+^ T cells (**C**, **D**), and CD21^low^ B cells (**E**, **F**) are shown. IFN-γ production and CD25 induction are shown, related to pro-Th1 response and cellular activation, respectively. For all panels, on the left side are shown plots displaying all individual animals, whereas on the right side are provided representative contour plots overlaying the signal obtained in unstimulated blood cells (black) with the signal obtained in cells exposed to bacteria (blue for activated cells derived from *Bos taurus* animals, red for activated cells derived from *Bos indicus* animals). Numbers indicate the percentage of IFN-γ^+^ lymphocytes obtained for the representative animal. The fold changes analysis of CD25 was determined by FCM with FlowJo 10.9.0. Cells from the individual *Bos taurus* and *Bos indicus* animals are represented by separate symbols; for stimulated samples, mean fluorescence intensity (MFI) values are normalized to that obtained with the reference point from the same animal. **G** Bacterial comparison for *Bos taurus* (blue histograms) and *Bos indicus* (red histograms) animals. Arrows indicate significant differences of IFN-γ production or CD25 induction following *Treponema phagedenis* compared with *Dichelobacter nodosus* exposure for a given cattle subspecies. Results are displayed for CD4^+^ T cells (left panel) and CD21^low^ B cells (right panel). Experimental conditions were compared using Mann–Whitney *U* tests (**A**–**F**) or repeated-measures one-way ANOVA followed by Tukey’s post hoc test (**G**). b, benign strain of *Dichelobacter nodosus*; v, virulent strain of *Dichelobacter nodosus*. Asterisks indicate significance levels. ^*^p < 0.05; ^**^p < 0.01; ^***^p < 0.001. Blue asterisks indicate significant higher levels in *Bos taurus* animals, whereas red asterisks indicate significant higher levels in *Bos indicus* animals. Assays were carried out at bovine body temperature of 38.5 °C.

Given that IFN-γ appeared to be a crucial cytokine in orchestrating the ex vivo response to the hoof microbes tested in this study, we next checked which specific subsets among *Bos taurus* PBMCs elicited very high amounts of IFN-γ. Thus, we assessed the responsiveness of T and B lymphocytes to both bacteria, especially by performing an in-depth investigation of activation state from subsets belonging to the adaptive arm of the immune system, as well of intracellular staining of IFN-γ. For clarity, data displayed in Figure 5 focus on CD4^+^ T cells (Figure 5A, B), CD8^+^ T cells (Figure 5C, D), and CD21^low^ B cells (Figure 5E, F), while the full panel is available in Additional files 7 and 8.

The response to *Treponema phagedenis* differed strongly between *Bos taurus* and *Bos indicus*. A few *Bos taurus* lymphocyte subsets displayed a remarkably high level of IFN-γ production. Specifically, the percentage of positive cells at low MOI of 0.1 was 18.0% for CD4^+^ T cells (Figure 5A) and 12.5% for CD21^low^ B cells (Figure 5E). Remarkably, the production of IFN-γ was even higher when bacteria were added at low MOI, suggesting that *Bos taurus* lymphocytes can respond even better at a very low threshold (Figure 5A and E).

In comparison, *Dichelobacter nodosus* proved to have little effect on IFN-γ production by lymphocytes—regardless of cattle subspecies investigated. Despite the low percentage of IFN-γ^+^ cells in *Bos taurus* animals, all those immune cell subsets appeared to be activated, as witnessed by the high upregulation of CD25 (Figure 5B, D, F). Cells from *Bos indicus* showed a similar pattern when exposed to *Dichelobacter nodosus* [i.e., almost no increased IFN-γ production despite high CD25 upregulation for CD4^+^ T cells (Figure 5B), CD8^+^ T cells (Figure 5D), and CD21^low^ B cells (Figure 5F)]. Additional file 8 shows that other T, B, and NK cells subsets made a moderate contribution to the whole IFN-γ secretion.

The differential ex vivo response to *Treponema phagedenis* between *Bos taurus* and *Bos indicus* was further confirmed when the analysis was done for a given cattle subspecies (Figure 5G). Indeed, for *Bos taurus* animals, readouts were significantly enhanced following stimulation by *Treponema phagedenis* (low for CD25 induction, very strong for IFN-γ secretion), whereas no consistent effect was seen in *Bos indicus* animals.

Finally, having previously shown that PBMC viability was significantly reduced for *Bos taurus* following bacterial infection (Figure 1E), we checked whether this viability loss contributed to the increased levels of cytokines such as IFN-γ (Figure 2, multiplex immunoassay; Figure 5, FCM). To this aim, we did a contextual gating strategy analysis omitting this time live/dead staining (“viable + nonviable cells”) and compared the results with those obtained from the classical analysis employing live/dead staining (“viable cells only”). For this, we considered the percentage of “all events” of IFN-γ^+^ CD4^+^ T cells, IFN-γ^+^ CD8^+^ T cells, and IFN-γ^+^ CD21^low^ B cells, calculated as follows: [number of events in the gated IFN-γ^+^ cell subset] / [number of events in gate P1, which contains all PBMC while excluding cell aggregates and debris]. Clearly, inclusion of dying cells in the new set of analysis done for “viable + nonviable cells” had no role in the percentage of all events of IFN^+^ cells (whether the CD4^+^ T cell, CD8^+^ T cell, or CD21^low^ B cell subset) (Additional file 9). This ruled out the possibility that the differences of IFN-γ levels between *Bos indicus* and *Bos taurus* animals could be attributable to overall higher cell death levels in the latter.

### The level of IFN-γ production against *Treponema phagedenis* and *Dichelobacter nodosus* moderately correlates with T and B lymphocyte activation states

**Figure 6:**
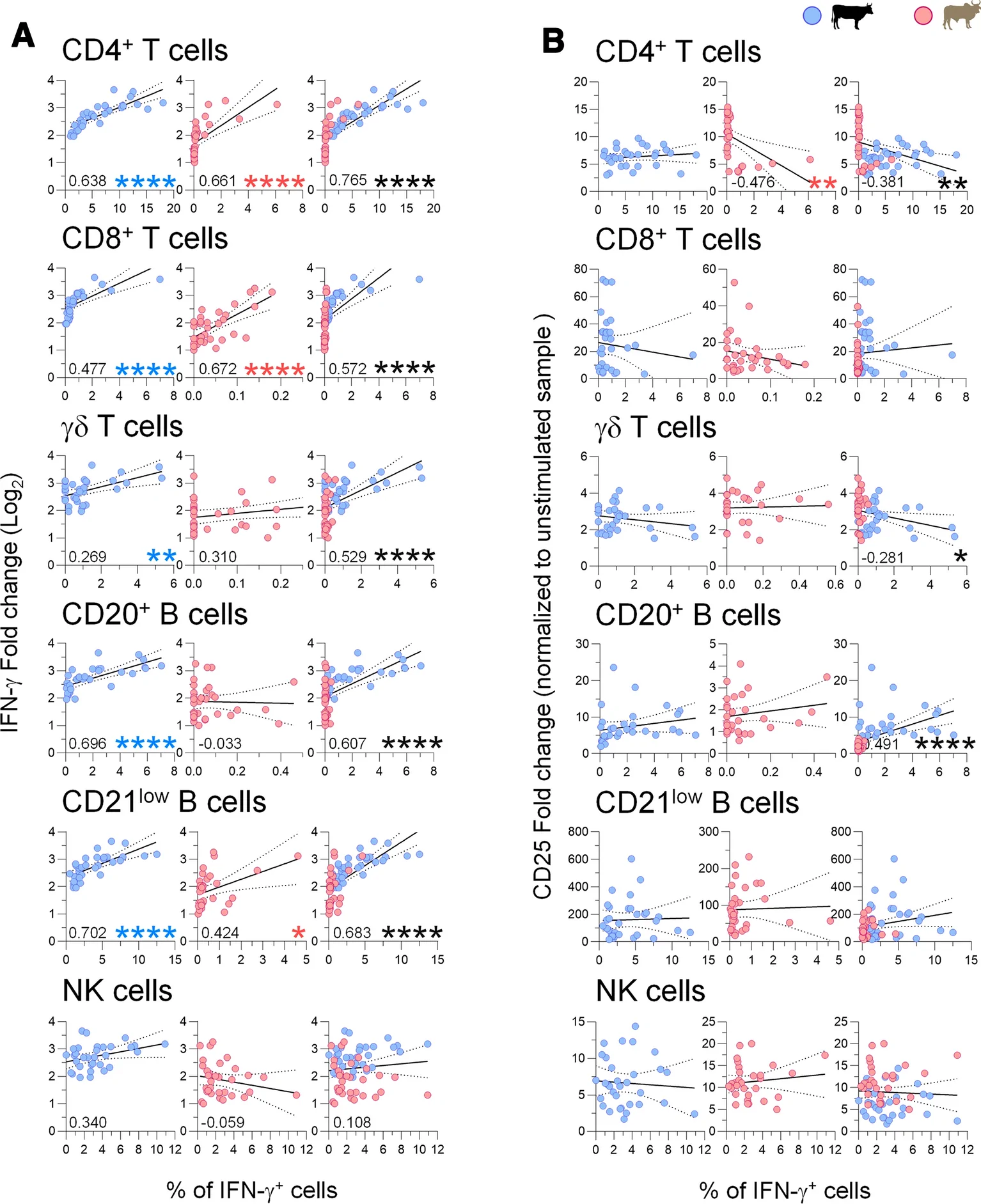
**The level of IFN-γ production against** ***Treponema phagedenis*** **and** ***Dichelobacter nodosus*** **poorly correlates with T and B lymphocyte activation states**. **A** Correlation coefficients obtained with secreted IFN-γ (multiplex immunoassay, fold-change) calculated as a function of the percentage of IFN-γ^+^ cells (FCM). Except for NK cells, significance was reached for all immune cell subsets. **B** Correlation coefficients obtained with T and B lymphocyte activation (FCM, induction of CD25) calculated as a function of percentage of IFN-γ^+^ cells (FCM). All correlations are organized as follows: on the left are shown the 32 plots in blue obtained for *Bos taurus* animals (eight individuals, four experimental conditions: *Treponema phagedenis* MOI = 1 and 0.1; *Dichelobacter nodosus* MOI = 1 and 0.1); in the middle are shown the 32 plots in red, obtained for *Bos indicus* animals (eight individuals, four experimental conditions: *Treponema phagedenis* MOI = 1 and 0.1; *Dichelobacter nodosus* MOI = 1 and 0.1); on the right are computed the plots obtained for all animals (*n* = 16, 64 plots). Associations were tested using the Pearson rank correlation test. Asterisks indicate significance levels. ^*^p < 0.05; ^**^p < 0.01; ^***^p < 0.001; ^****^p < 0.0001. Blue asterisks indicate significant higher levels in *Bos taurus* animals, whereas red asterisks indicate significant higher levels in *Bos indicus* animals. Assays were carried out at bovine body temperature of 38.5 °C.

To corroborate the above results, we calculated percentages of IFN-γ^+^ lymphocytes subsets (multiparameter FCM) as a function of IFN-γ concentration in the supernatant (multiplex immunoassay). As observed above for correlation done with APC subsets (Figure 4), data related to *Bos taurus* animals (blue spots) tend to cluster away from data obtained from *Bos indicus* animals (red spots), providing further evidence that the ex vivo response broadly differs between the two cattle subspecies. When data sets from both cattle subspecies were overlayed, a strong association was found for all tested immune cell subsets, apart from NK cells, which was relatively intriguing given that this specific subset was the high responder against *Mycoplasmopsis bovis* [28] (Figure 6A). Interestingly, the percentage of IFN-γ^+^ cells was associated with activation levels (CD25 expression levels) for B cells (positive correlation) and to a lesser extent CD4^+^ T cells (negative correlation). Nevertheless, associations found between IFN-γ production and activation of adaptive immune cell subsets were at best moderate (Figure 6B), when compared with what was observed above with nonclassical monocytes and cDC2s (Figure 4).

### Matrix metalloproteinase secretion by immune cells after infection with *Treponema phagedenis* and *Dichelobacter nodosus*

**Figure 7:**
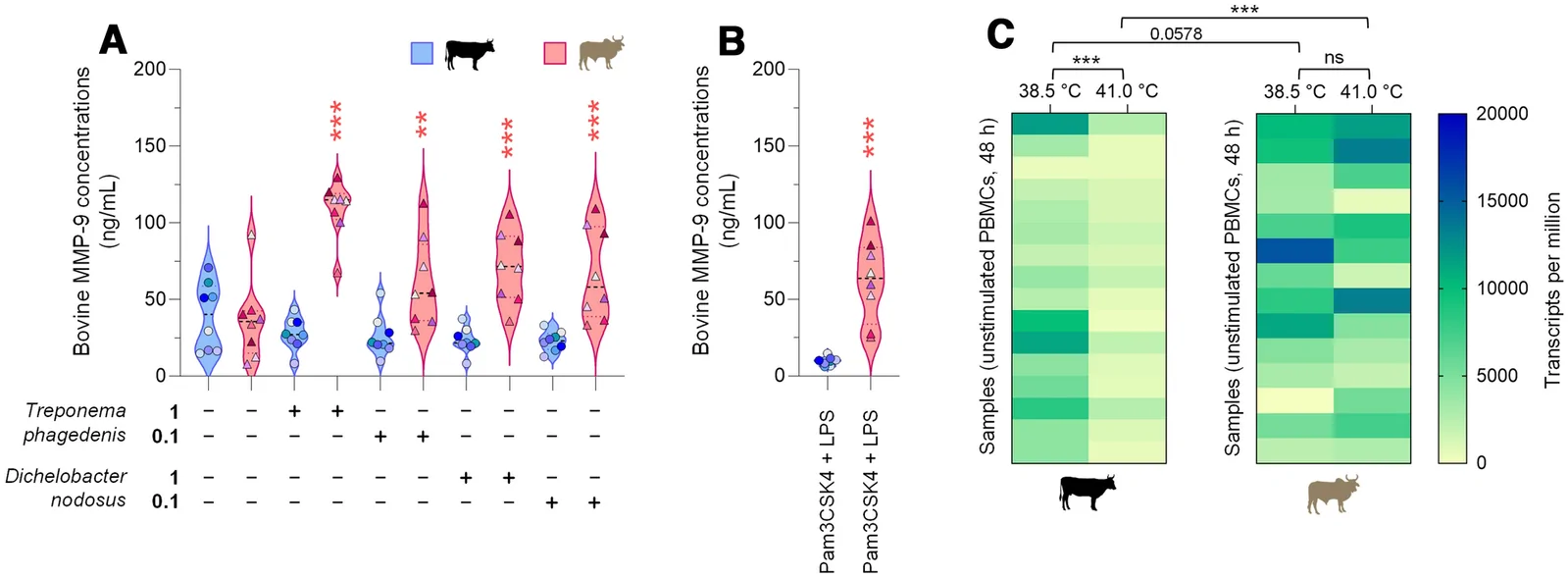
**Matrix metalloproteinase secretion by immune cells after infection with** ***Treponema phagedenis*** **and** ***Dichelobacter nodosus***. **A**, **B** MMP-9 production quantified by ELISA (ng/mL). **A** PBMCs from eight individual animals per group, whether *Bos taurus* or *Bos indicus*, were either let for 16 h unstimulated (reference points for the assay) or stimulated with *Dichelobacter nodosus* (MOI = 1 and 0.1) or *Treponema phagedenis* (MOI = 1 and 0.1). **B** PBMCs from eight individual animals per group, whether *Bos taurus* or *Bos indicus*, were stimulated with synthetic agonists (Pam3CSK4 + LPS, 10 μg/mL). **C** Number of detected MMP-9 transcripts per million from RNA extracted from unstimulated PBMCs at *T* = 48 h (*N*_*Bos taurus*_ = 16, *N*_*Bos indicus*_ = 14). Those transcriptomic data are reused from a recent study where bulk RNA-Seq was performed in PBMCs derived from both *Bos taurus* and *Bos indicus* animals [29]. Experimental conditions were compared using Mann–Whitney *U* tests [**A**, **B** and **C** (interbreed comparison)] or nonparametric paired Wilcoxon tests [**C** (temperature comparison)]. Asterisks indicate significance levels. ^*^p < 0.05; ^**^p < 0.01; ^***^p < 0.001. Red asterisks indicate significant higher levels in *Bos indicus* animals. Assays were carried out at bovine body temperature of 38.5 °C.

Having shown the strong ex vivo inflammatory response of *Bos taurus* animals to *Treponema phagedenis*, we assessed the level of matrix metalloproteinases (MMP), and more specifically MMP-9. Indeed, while MMP-9 is essential for neutrophil migration, pathogen clearance, and tissue repair, its persistent activity can lead to a pathogenic response driving chronic inflammation, which would fit with our findings in *Bos taurus* individuals. However, this experiment revealed an unexpected observation. MMP-9 levels within the supernatants were high for cells derived from *Bos indicus* animals, in particular when exposed to *Treponema phagedenis* at high MOI (Figure 7A), which does not match the readouts obtained for secreted pro-inflammatory cytokines (Figure 2A–C). Thus, these intriguing results prompted us to examine the intrinsic capacity of PBMCs from both cattle subspecies to secrete MMP-9 in response to synthetic agonists. Stimulation by TLR ligands almost abrogated the background levels detected in *Bos taurus* individuals. In contrast, in *Bos indicus* animals, a strong—but variable—secretion of MMP-9 in the supernatant of stimulated cells was observed (Figure 7B). From this, it was clear that, depending on their subspecies origin, primary blood cells respond to stimulation by orchestrating a differential innate immune response. To confirm that MMP-9 levels can differ between the two cattle subspecies at the initial stage of the immune response, we datamined already published transcriptomic data from a recent study employing bulk RNA-Seq in unstimulated PBMCs from *Bos taurus* and *Bos indicus* animals, either at body temperature of 38.5 °C or at fever-like temperature of 41.0 °C [29]. Certainly, the number of MMP-9 transcripts per million strongly dropped down in *Bos taurus* when temperature rose to 41.0 °C, whereas transcript numbers remained high in *Bos indicus* animals (Figure 7C).

## Discussion

BDD is a disease that was first described in 1974 [35] and since then has been recognized endemic in many parts of the world. It affects mainly dairy cattle and was reported in Switzerland over the past 20 years. This painful bovine hoof disease is polymicrobial but clearly linked to *Treponema phagedenis* among other *Treponema* species such as *Treponema medium* and *Treponema pedis* [36]. *Treponema phagedenis* strains isolated from BDD lesions differ in their genetic composition to human strains [31, 37]. Pathogenesis of BDD is characterized by local inflammation of the skin of the heel bulb but remains poorly understood regarding the sequence of events and the microbes and their traits driving the inflammation. Hence, effective treatments are lacking to date. In a recent study we reported a low prevalence of BDD-associated *Treponema* sp. in cattle of *Bos indicus* origin. We did not detect *Treponema phagedenis* in heel bulb swabs and punch biopsies in Swiss *Bos indicus* cattle, while 35% of Swiss *Bos taurus* cattle investigated were found positive [17]. Brazilian crossbred cattle of Holstein × Gyr kept at pasture have been reported to be susceptible to BDD and different *Treponema* species have been detected in most lesions, while *Dichelobacter nodosus* has been detected in only about half of the lesions investigated [38]. The present study took advantage of a recently established blood-based ex vivo platform to investigate host–microbe interactions with focus on immune cells [28]. Switzerland houses several hundred *Bos indicus* cattle that are kept under similar housing conditions as local *Bos taurus*, enabling us to compare their immune responses to different microbes. Our 16 animals encompassed only 2 male animals, which is at the lower side. But since BDD affects mainly cows, we are confident that the data presented here are very relevant. Our study provides results in line with the notion that the high prevalence of BDD in *Bos taurus* corresponds to a stronger pro-Th1/proinflammatory ex vivo response to *Treponema phagedenis* (high level of secreted IFN-γ, IL-1α, IL-1β, and TNF-α), underpinning our recent findings showing a solid link between cattle breed, over-exuberant immune response, and susceptibility to disease caused by emerging vector-borne viruses in Europe [29].

There are some limitations to the present work. First the genetic diversity of Swiss *Bos indicus*, although quite diverse [17], does not represent the entire *Bos indicus* population; this is also true for the *Bos taurus* cattle enrolled in this study. Moreover, BDD is a polymicrobial disease, and we investigated only *Treponema phagedenis* as a BDD-causing agent apart from *Dichelobacter nodosus*, with the latter not strongly correlated with BDD [13, 36, 39]. However, in our previous study, we could show that *Treponema phagedenis* was significantly more often found in animals with BDD lesions than in visibly healthy animals, affirming that this bacterium is contributing to BDD and is worth investigating [32]. Furthermore, we focused our work on PBMCs, although it is now established that other immune cell subsets such as neutrophils are also critical. They produce lactotransferrin and cathelicidins in active (M2) BDD lesions, exerting antimicrobial activity [40], which appears to be insufficient to prevent further inflammatory relapses [41]. The undesired effect of the joint response driven by neutrophils and monocytes/macrophages impedes ulcerative lesion healing in active BDD [42]. In addition, fibroblasts, which are important cells of the bovine foot skin, were not considered in this study [43]. They maintain skin structure, whose morphology differs among cattle breeds, with *Bos indicus* having thicker layer favoring tolerance to heat and impeding dermis accumulation [44]. Finally, we worked with only one strain of *Treponema phagedenis*, which was isolated from an active BDD lesion in 2018 [31].

We compared cattle subspecies with a different genetic background and purpose. *Bos taurus* are highly optimized breeds subjected to greater metabolic stress due to their dairy use, whereas *Bos indicus* studied here has a high genomic diversity and are mainly used for meat production. We characterized the immune system of both cattle groups in investigating the background cytokine levels in the serum, as well as their intrinsic capacity to respond to synthetic agonists such as TLR-2 and TLR-4. Importantly, we ran all immunological assays at bovine body temperature of 38.5 °C since we showed recently that working at ruminant body temperature provides more meaningful data than working at 37 °C [28, 30]. Overall, we found that their respective immune parameters were largely comparable, albeit with some few but noticeable differences, such as IP-10 background levels in serum, which was higher in *Bos indicus* animals or IL-1-β secretion following PBMC stimulation by TLR ligands, which was higher in *Bos taurus* animals. From this, we showed that the immune system of all animals enrolled in the study—regardless of *Bos taurus* or *Bos indicus*—(i) was resting in vivo and (ii) displayed a robust and comparable potential to respond to potent synthetic immunostimulators. Of note, other possible environmental factors influencing our assays, such as housing and foot hygiene including hoof trimming [45, 46], have not been taken into account. However, both farming systems are tightly controlled by Swiss authorities and had no reported hygiene issues, making this unlikely to affect our immunological parameters. Therefore, our comparisons of the immune responses to *Treponema phagedenis* or *Dichelobacter nodosus* were relevant.

Classical CD14^high^CD16^low^ monocytes/macrophages are recruited and persist in active BDD lesions [42]. This study found their activation level significantly increased in *Bos taurus* individuals as shown by respective CD25 and CCR7 upregulation and a strong MHC-II drop down (Additional file 5). But what really raised our interest was the role of nonclassical CD14^low^CD16^high^ monocytes, which were previously shown in *Bos indicus* animals to act against vector-borne viruses such as “hyper-presenting cells,” witnessed by MHC-II higher levels but non-consistent CD25 and CCR7 upregulation [29]. We found that this putative function also occurred in response to hoof bacteria, where infection by *Treponema phagedenis* led to significantly higher MHC-II levels and lower levels of CD25/CCR7. Overall, this highlights the potential protecting role of nonclassical monocytes/macrophages in *Bos indicus* animals in fighting microbial agents. To get more insight, we aim in future studies to sort these specific immune cell subsets following infection by microbes such as vector-borne viruses or *Treponema phagedenis* and investigate whether transcriptomic profiles differ between the two cattle subspecies, postulating that we will pinpoint key gene contributors conferring immunity to pathogens.

Another major finding was the differential response of *Bos taurus* and *Bos indicus* animals related to the CD4^+^ T cell subset following infection by *Treponema phagedenis*. Indeed, in *Bos taurus*, CD4^+^ T cells displayed a massive IFN-γ secretion but moderate CD25 upregulation, whereas in *Bos indicus*, they produced at best moderate levels of IFN-γ but showed enhanced CD25 upregulation. This observation roughly corroborates recent findings employing vector-borne viruses, where CD4^+^ T cells from *Bos indicus* animals also displayed enhanced levels of CD25 expression [29]. Herein, what was particularly impressive was the huge IFN-γ response to *Treponema phagedenis* that happened in all tested *Bos taurus* animals, despite the fact that only two of them were previously infected, as witnessed by the presence of specific antibodies for this pathogen in their serum (Additional file 1). Consequently, the massive IFN-γ production was most likely a bystander effect—activation of nonspecific T cells without cognate antigens—as this phenomenon happens during bacterial infection [28, 47, 48]. Another study supported the notion that CD4^+^ T cells are inefficient in *Treponema* clearance in infected *Bos taurus* animals: unlike CD8^+^ and γδ T cells, they lack responsiveness when restimulated by treponemal antigens [49]. For all those reasons—and as stated previously for nonclassical monocytes—we are convinced that proceeding to the sort of CD4^+^ T cells following infection by vector-borne viruses or *Treponema phagedenis*, and comparing the respective transcriptomic profiles of both cattle subspecies, will be of great help in identifying players conferring efficient pathogen clearance or promoting undesired immunopathogenesis.

Given that the strong inflammatory response to *Treponema phagedenis* is a trademark of the innate ex vivo response in *Bos taurus* animals and matrix metalloproteinase-9 (MMP-9) has been detected at critically elevated levels in chronic and inflamed wounds [50, 51], we expected to find elevated concentrations of this endopeptidase in the supernatant of their infected PBMCs. This expectation was due to the aforementioned high level of IFN-γ, known to act as a critical mediator of MMP-9 production [52]. Variable and comparable levels of MMP-9 were detected in both bovine subspecies in unstimulated PBMCs, but infection with microbes in this study led to an unexpected observation. MMP-9 levels were significantly stronger in *Bos indicus* animals, while they were almost abrogated in *Bos taurus* animals. At first glance, this seemed to conflict radically with a recent study from Lahiri et al. investigating *Bos taurus*-like cattle suffering from BDD with active M2 lesions, which exhibited both increased immune cell infiltration and high MMP-9 activity in their foot skin [41]. Nevertheless, one must consider the sequential and dualistic activity of MMP-9. On the one hand, transient activity is indispensable for effective host defense, notably by processing chemokines and TNF-α, which facilitates tissue repair as well as neutrophil and macrophage extravasation due to cleavage of type IV collagen, laminin, and other components from the extracellular matrix [53]. On the other hand, MMP-9-sustained expression drives chronic inflammatory disorders by perpetuating undesired leukocyte infiltration and tissue injury, as seen in rheumatoid arthritis, inflammatory bowel disease, and chronic pulmonary disorders [54, 55]. Consequently, in the specific context of BDD, moderate levels of proinflammatory cytokines might be beneficial in *Bos indicus* cattle due to beneficial remodeling of the extracellular matrix mediated by MMP-9, which allows better diffusion toward the site of infection. On the contrary, higher levels of proinflammatory cytokines might be required in infected *Bos taurus* individuals, due to the physical barrier that represents less remodulated extracellular matrix, possibly leading to sustained inflammatory milieu. However, we are aware that the use of PBMC, as every other model system does, has limitations and does not constitute a particularly appropriate model to evaluate long-term outcomes of infections, as observed in BDD. Effectively, the development of immunological memory implies DC migration to draining lymph nodes (LNs) followed by antigen-specific T and B cell recruitment to inflamed BDD lesions, and this could not be evaluated in the present study. Therefore, future work is needed to clarify the role of MMP-9 in different stages of pathogenesis, notably enzymatic activity, cellular source, and functional effects on bacterial clearance.

### Concluding remarks

When initiating this study, it was known that *Bos indicus* cattle are more resistant to colonization by *Treponema* spp. than *Bos taurus* ones. However, the link between their higher resistance and a less pronounced inflammatory response to *Treponema phagedenis* was absent. We did not provide causal evidence but rather an association between a strong inflammatory ex vivo immune response of *Bos taurus* cattle to *Treponema phagedenis* and known higher susceptibility to BDD of this cattle subspecies. This is in line with previous findings regarding immune responses to emerging vector-borne viruses [29]. Finally, the present work showed a significant stronger induction of MMP-9 by PBMCs exposed to *Treponema phagedenis* of *Bos indicus* versus *Bos taurus* cattle. While MMP-9 has been shown to be a contributing factor to BDD in active or chronic lesions its high expression at the onset of an infection might foster clearance of the invading pathogens with efficient local leukocyte recruitment.

## Supplementary Information

Additional file 1. Characteristics of animals enrolled in the study. * Detection of antibodies against *Treponema phagedenis* in the serum of animals.

**Additional file 2. Antibodies used in the study.**

**Additional file 3. FCM gating strategy for immune cell identification. **(**A**) Example of gating strategy for multiparameter FCM analysis of monocytes. Gating strategy following multiparameter FCM staining used Abs against CD172a, CD16, CD14, CD13, CD4 and CD11c. A primary gate (P1) was set on FSC-A versus SSC-A. Then, the dead cell population (positive for Live/Dead) was excluded, followed by a FSC-H/FSC-A contour plot (to exclude doublets from the analysis). Next, we defined among this population the cells highly positive for CD172a marker (CD172a^high^ cells), positive for CD13 and CD11c, and then negative for CD4. From this, classical monocytes (c.Mono) were gated as CD14^+^CD16^-^, intermediate monocytes (int.Mono) as CD14^+^CD16^+^, and nonclassical monocytes (nc.Mono) as CD14^-^CD16^+^. Maturation was then evaluated based on MFI of surface expression for MHC-II, CD25 and CCR7. (**B**) Example of gating strategy for multiparameter FCM analysis of DCs. Gating strategy following multiparameter FCM staining used Abs against MHC-II, CD172a, CD16, CD14, CD13, CD4 and CD11c. A primary gate (P1) was set on FSC-A versus SSC-A. Then, the dead cell population (positive for Live/Dead) was excluded, followed by a FSC-H/FSC-A contour plot (to exclude doublets from the analysis). Among these cells, we defined the population positive for MHC-II and CD172a (low and intermediate only). Then, we defined among this population the cells negative for both CD14 and CD16 markers. Then, we defined among this population either the cells positive for CD13 and negative for CD4 markers (pre-cDC1s), either cells negative for CD13 and positive for CD4 markers (pre-pDCs), either cells negative for both (pre-cDC2s). Next, we considered CD11c marker to gate cDC1s and cDC2s (respectively pre-cDC1s and pre-cDC2s positive for CD11c), as well as pDCs (pre-pDCs negative for CD11c). Maturation was evaluated based on the MFI of surface expression for MHC-II, CD25 and CCR7. (**C**) Example of gating strategy for multiparameter FCM analysis to identify T cells. Gating strategy used Abs against CD3, CD4, CD8, CD25, WC1, CD44, CD62L, IL-4, IFN-γ and IL-17. A primary gate (P1) was set on FSC-A versus SSC-A encompassing both small and large cells. Then, the dead cell population (positive for Live/Dead) was excluded, followed by a FSC-H/FSC-A contour plot (to exclude doublets from the analysis). Then, we considered cells positive for CD3, and defined among this population the cells negative for both CD4 and CD8 markers. Among these cells, γδ T cells were defined as the WC1^+^ cells, and the maturation was evaluated based on CD25 expression, whereas activation was evaluated on the basis of IFN-γ and/or IL-17. Alternatively, we defined among the CD3^+^ population either the cells positive for CD4 and negative for CD8 markers (CD4^+^ T cells), either cells positive for CD8 and negative for CD4 markers (CD8^+^ T cells). The maturation was evaluated based on CD25 expression, as well as percentage of central (CD62L^+^CD44^+^) or effector (CD62L^-^CD44^+^) memory cells. and on the basis of IFN-γ, IL-4 and/or IL-17. (**D**) Example of gating strategy for multiparameter FCM analysis of NK and B cells. Gating strategy following multiparameter FCM staining used Abs against CD3, CD20, CD21 and CD335. A primary gate (P1) was set on FSC-A versus SSC-A. Then, the dead cell population (positive for Live/Dead) was excluded, followed by a FSC-H/FSC-A contour plot (to exclude doublets from the analysis). Next, we defined among this population the cells negative for CD3 marker. Then, we defined among this population the cells negative for both CD20 and CD21 markers. From this, NK cells were gated as CD335^+^ cells. Alternatively, we defined among the population negative for CD335 either the cells positive for CD20 (CD20^+^ B cells), either the cells negative for CD20 marker and positive for CD21 (CD21^low^ and CD21^high^ B cells). The maturation was evaluated based on CD25 expression, as well as on the basis of IFN-γ, IL-4, TNF-α and/or IL-17.

**Additional file 4. Infection of primary peripheral blood mononuclear cells by *Dichelobacter nodosus*** **(virulent for sheep)**. PBMCs from eight individual animals per group were either unstimulated or stimulated with virulent strain of *Dichelobacter nodosus* (DNT71KR isolate). (**A-C**) Interbreed comparison of cytokine induction for *Dichelobacter nodosus*. Infections were run at ruminant body temperature (38.5 °C). A single measurement was done per sample tested, and each symbol represents an individual animal. (**A**) Heat map shows log_2_-fold changes in concentration of 15 cytokines/chemokines. For a given cytokine/chemokine, normalization was as follows: [concentration for a given animal]/[average concentration of reference points]. (**B**) Radar plots showing the mean of eight animals of log_2_-fold changes in concentration. (**C**) The concentrations (pg/mL) of the major proinflammatory cytokines following primary blood cells following exposure to *Dichelobacter nodosus* are presented. The Violin plots show the values obtained from the eight animals. (**D-F**) In these panels data obtained for nonclassical monocytes and cDC2s are shown. Three activation/maturation markers were considered: MHC-II (**D**), CD25 (**E**) and CCR7 (**F**). For each panel, on the left side are shown graphs displaying all individual animals. (**G-H**) In these panels data obtained for CD4^+^ T cells, CD8^+^ T cells and CD21^low^ B cells are shown. Two activation/maturation readouts were considered: IFN-γ production (**G**) and CD25 (**H**). (**D**-**H**) For each panel, on the left side are shown graphs displaying all individual animals. The fold changes analysis of activation/maturation markers was determined by FCM with FlowJo 10.9.0. Cells from the individual *Bos taurus* and *Bos indicus*-like cattle are represented by separate symbols; for stimulated samples, mean fluorescence intensity (MFI) values are normalized to that obtained with the reference point from the same animal. On the right side are provided representative contour plots the overlaying signal obtained in unstimulated blood cells (black) with the signal obtained in cells exposed to bacteria (blue for activated cells derived from *Bos taurus* animals, red for activated cells derived from *Bos indicus* animals). Experimental conditions were compared using Mann-Whitney *U* tests. Asterisks indicate significance levels. *, *p* < 0.05; **, *p* < 0.01; ***, *p* < 0.001. Assays were carried out at bovine body temperature of 38.5 °C.

**Additional file 5. Response of antigen-presenting cells toward** ***Treponema phagedenis***. PBMCs from eight individual animals per group, whether *Bos taurus* or *Bos indicus*, were either left for 20 h unstimulated (reference points for the assay) or stimulated with *Treponema phagedenis* at high MOI of 1 or at low MOI of 0.1. The fold changes analysis of activation/maturation markers was determined by FCM with FlowJo 10.9.0. Cells from the individual *Bos taurus* and *Bos indicus* animals are represented by separate symbols; for stimulated samples, mean fluorescence intensity (MFI) values for MHC-II, CD25 and CCR7 are normalized to that obtained with the reference point from the same animal. Results obtained for classical monocyte, intermediate monocyte, nonclassical monocyte, Type 1 conventional DC, Type 2 conventional DC and plasmacytoid DC subsets. Experimental conditions were compared using Mann-Whitney *U* tests. Asterisks indicate significance levels. *, *p* < 0.05; **, *p* < 0.01; ***, *p* < 0.001. Blue asterisks indicate significant higher levels in *Bos taurus* animals, whereas red asterisks indicate significant higher levels in *Bos indicus* animals. Assays were carried out at bovine body temperature of 38.5 °C.

**Additional file 6. Response of antigen-presenting cells toward *Dichelobacter nodosus***. PBMCs from eight individual animals per group, whether *Bos taurus* or *Bos indicus*, were either left for 20 h unstimulated (reference points for the assay) or stimulated with *Dichelobacter nodosus* at high MOI of 1 or at low MOI of 0.1. The fold changes analysis of activation/maturation markers was determined by FCM with FlowJo 10.9.0. Cells from the individual *Bos taurus* and *Bos indicus* animals are represented by separate symbols; for stimulated samples, mean fluorescence intensity (MFI) values for MHC-II, CD25 and CCR7 are normalized to that obtained with the reference point from the same animal. Results obtained for classical monocyte, intermediate monocyte, nonclassical monocyte, Type 1 conventional DC, Type 2 conventional DC and plasmacytoid DC subsets. Experimental conditions were compared using Mann-Whitney *U* tests. Asterisks indicate significance levels. *, *p* < 0.05; **, *p* < 0.01; ***, *p* < 0.001. Blue asterisks indicate significant higher levels in *Bos taurus* animals, whereas red asterisks indicate significant higher levels in *Bos indicus* animals. Assays were carried out at bovine body temperature of 38.5 °C.

**Additional file 7. T cell, B cell and NK cell response to *Treponema phagedenis***. PBMCs from eight individual animals per group, whether *Bos taurus* or *Bos indicus*, were either left for 20 h unstimulated (reference points for the assay) or stimulated with *Treponema phagedenis* at High (1) or Low (0.1) MOI. The fold changes analysis of activation/maturation markers was determined by FCM with FlowJo 10.9.0. Cells from the individual *Bos taurus* and *Bos indicus* animals are represented by separate symbols. (Left side) For stimulated samples, mean fluorescence intensity (MFI) values for CD25 are normalized to that obtained with the reference point from the same animal. (Right side) Percentage of IFN-γ^+^ cells. Results obtained for CD4^+^ T cell, CD8^+^ T cell, γδ T cell, CD20^+^ B cells, CD21^low^ B cells and NK cell subsets. Experimental conditions were compared using Mann-Whitney *U* tests. Asterisks indicate significance levels. *, *p* < 0.05; **, *p* < 0.01; ***, *p* < 0.001. Blue asterisks indicate significant higher levels in *Bos taurus* animals, whereas red asterisks indicate significant higher levels in *Bos indicus* animals. Assays were carried out at bovine body temperature of 38.5 °C.

**Additional file 8. T cell, B cell and NK cell response to *Dichelobacter nodosus***. PBMCs from eight individual animals per group, whether *Bos taurus* or *Bos indicus*, were either left for 20 h unstimulated (reference points for the assay) or stimulated with *Dichelobacter nodosus* at High (1) or Low (0.1) MOI. The fold changes analysis of activation/maturation markers was determined by FCM with FlowJo 10.9.0. Cells from the individual *Bos taurus* and *Bos indicus* animals are represented by separate symbols. (Left side) For stimulated samples, mean fluorescence intensity (MFI) values for CD25 are normalized to that obtained with the reference point from the same animal. (Right side) Percentage of IFN-γ^+^ cells. Results obtained for CD4^+^ T cell, CD8^+^ T cell, γδ T cell, CD20^+^ B cells, CD21^low^ B cells and NK cell subsets. Experimental conditions were compared using Mann-Whitney *U* tests. Asterisks indicate significance levels. *, *p* < 0.05; **, *p* < 0.01; ***, *p* < 0.001. Blue asterisks indicate significant higher levels in *Bos taurus* animals, whereas red asterisks indicate significant higher levels in *Bos indicus* animals. Assays were carried out at bovine body temperature of 38.5 °C.

**Additional file 9**. **Absence of link between reduced PBMC viability and increased number of IFN-γ**^**+**^ **CD4**^**+**^ **T cells following bacterial infection in** ***Bos taurus*** **animals**. (A) The top displays the classical gating strategy used for the whole study (“Viable cells only”, as shown in Additional file 3C) and the bottom shows the contextual gating strategy omitting Live/Dead staining (“Viable + nonviable cells”). In this specific example (*Bos taurus* PBMCs infected by *Treponema phagedenis* at low MOI of 0.1), it clearly shows that the quantified number of IFN-γ^+^ CD4^+^ T cells is almost unchanged whether we considered “Viable cells only” (*n* = 2429) or “Viable + nonviable cells” (*n* = 2444). (B) PBMCs from eight individual animals per group, whether *Bos taurus* or *Bos indicus*, were stimulated with *Treponema phagedenis*. Numbers indicate the percentage of total events of IFN-γ^+^ CD4^+^ and CD8^+^ T cells, and CD21^low^ B cells, obtained by calculating the ratio [number of events in the gated IFN-γ^+^ cell subset] to [number of events in P1, gate comprising all events with the exception cell aggregates and debris]. The values obtained with “Viable cells only” analysis (blue bars) and “Viable and nonviable cells” analysis (hatched blue bars) were almost identical for the three investigated cell subsets. Animals are represented by separate symbols. Assays were carried out at bovine body temperature of 38.5 °C.

## Data Availability

No datasets were generated or analyzed during the current study.

## References

[1] Greenspoon L, Krieger E, Sender R, Rosenberg Y, Bar-On YM, Moran U, Antman T, Meiri S, Roll U, Noor E, Milo R (2023) The global biomass of wild mammals. Proc Natl Acad Sci U S A 120:e2204892120 10.1073/pnas.2204892120 PMC10013851 36848563

[2] Cha E, Hertl JA, Bar D, Grohn YT (2010) The cost of different types of lameness in dairy cows calculated by dynamic programming. Prev Vet Med 97:1–8 10.1016/j.prevetmed.2010.07.011 20801533

[3] Robcis R, Ferchiou A, Berrada M, Raboisson D (2023) Management of digital dermatitis in dairy herds: optimization and time allocation. Animals (Basel) 13:1988 10.3390/ani13121988 PMC10294801 37370498

[4] Solano L, Barkema HW, Jacobs C, Orsel K (2017) Validation of the M-stage scoring system for digital dermatitis on dairy cows in the milking parlor. J Dairy Sci 100:1592–1603 10.3168/jds.2016-11365 27889123

[5] Solano L, Barkema HW, Mason S, Pajor EA, LeBlanc SJ, Orsel K (2016) Prevalence and distribution of foot lesions in dairy cattle in Alberta, Canada. J Dairy Sci 99:6828–6841 10.3168/jds.2016-10941 27236761

[6] Dopfer D, Koopmans A, Meijer FA, Szakall I, Schukken YH, Klee W, Bosma RB, Cornelisse JL, van Asten AJ, ter Huurne AA (1997) Histological and bacteriological evaluation of digital dermatitis in cattle, with special reference to spirochaetes and *Campylobacter faecalis*. Vet Rec 140:620–623 10.1136/vr.140.24.620 9228692

[7] Watts KM, Lahiri P, Arrazuria R, De Buck J, Knight CG, Orsel K, Barkema HW, Cobo ER (2020) Oxytetracycline reduces inflammation and treponeme burden whereas vitamin D(3) promotes beta-defensin expression in bovine infectious digital dermatitis. Cell Tissue Res 379:337–348 10.1007/s00441-019-03082-y 31410630

[8] Holzhauer M, Ploegmakers-van Deventer R, Smits D, Swart W (2017) Comparing the curative efficacy of topical treatment with thiamphenicol and oxytetracycline on digital dermatitis lesions in dairy cattle. Vet Rec 180:500 10.1136/vr.103758 28465327

[9] Beninger C, Naqvi SA, Naushad S, Orsel K, Luby C, Derakhshani H, Khafipour E, De Buck J (2018) Associations between digital dermatitis lesion grades in dairy cattle and the quantities of four *Treponema* species. Vet Res 49:111 10.1186/s13567-018-0605-z PMC6206660 30373670

[10] Evans NJ, Brown JM, Demirkan I, Birtles R, Hart CA, Carter SD (2009) In vitro susceptibility of bovine digital dermatitis associated spirochaetes to antimicrobial agents. Vet Microbiol 136:115–120 10.1016/j.vetmic.2008.10.015 19081208

[11] Krull AC, Shearer JK, Gorden PJ, Cooper VL, Phillips GJ, Plummer PJ (2014) Deep sequencing analysis reveals temporal microbiota changes associated with development of bovine digital dermatitis. Infect Immun 82:3359–3373 10.1128/IAI.02077-14 PMC4136199 24866801

[12] Walker RL, Read DH, Loretz KJ, Nordhausen RW (1995) Spirochetes isolated from dairy cattle with papillomatous digital dermatitis and interdigital dermatitis. Vet Microbiol 47:343–355 10.1016/0378-1135(95)00114-x 8748549

[13] Rasmussen M, Capion N, Klitgaard K, Rogdo T, Fjeldaas T, Boye M, Jensen TK (2012) Bovine digital dermatitis: possible pathogenic consortium consisting of *Dichelobacter nodosus* and multiple *Treponema* species. Vet Microbiol 160:151–161 10.1016/j.vetmic.2012.05.018 22698300

[14] Sullivan LE, Evans NJ, Blowey RW, Grove-White DH, Clegg SR, Duncan JS, Carter SD (2015) A molecular epidemiology of treponemes in beef cattle digital dermatitis lesions and comparative analyses with sheep contagious ovine digital dermatitis and dairy cattle digital dermatitis lesions. Vet Microbiol 178:77–87 10.1016/j.vetmic.2015.04.011 25937315

[15] Alsaaod M, Locher I, Jores J, Grimm P, Brodard I, Steiner A, Kuhnert P (2019) Detection of specific *Treponema* species and *Dichelobacter nodosus* from digital dermatitis (Mortellaro’s disease) lesions in Swiss cattle. Schweiz Arch Tierheilkd 161:207–215 10.17236/sat00201 30942188

[16] Arduser F, Moore-Jones G, Gobeli Brawand S, Durr S, Steiner A, Ryser-Degiorgis MP, Zanolari P (2020) *Dichelobacter nodosus* in sheep, cattle, goats and South American camelids in Switzerland—assessing prevalence in potential hosts in order to design targeted disease control measures. Prev Vet Med 178:104688 10.1016/j.prevetmed.2019.05.001 31109750

[17] Kuhnert P, Loosli N, Brodard I, Lindtke D, Jores J (2024) Resistance of zebu cattle (*Bos indicus*) to colonization by major ruminant hoof pathogens. Vet Microbiol 296:110184 10.1016/j.vetmic.2024.110184 38996749

[18] Stauble A, Steiner A, Frey J, Kuhnert P (2014) Simultaneous detection and discrimination of virulent and benign *Dichelobacter nodosus* in sheep of flocks affected by foot rot and in clinically healthy flocks by competitive real-time PCR. J Clin Microbiol 52:1228–1231 10.1128/JCM.03485-13 PMC3993488 24452162

[19] Bonfiglio S, Ginja C, De Gaetano A, Achilli A, Olivieri A, Colli L, Tesfaye K, Agha SH, Gama LT, Cattonaro F, Penedo MC, Ajmone-Marsan P, Torroni A, Ferretti L (2012) Origin and spread of *Bos taurus*: new clues from mitochondrial genomes belonging to haplogroup T1. PLoS One 7:e38601 10.1371/journal.pone.0038601 PMC3369859 22685589

[20] Edwards CJ, Bollongino R, Scheu A, Chamberlain A, Tresset A, Vigne JD, Baird JF, Larson G, Ho SY, Heupink TH, Shapiro B, Freeman AR, Thomas MG, Arbogast RM, Arndt B, Bartosiewicz L, Benecke N, Budja M, Chaix L, Choyke AM, Coqueugniot E, Dohle HJ, Goldner H, Hartz S, Helmer D, Herzig B, Hongo H, Mashkour M, Ozdogan M, Pucher E, Roth G, Schade-Lindig S, Schmolcke U, Schulting RJ, Stephan E, Uerpmann HP, Voros I, Voytek B, Bradley DG, Burger J (2007) Mitochondrial DNA analysis shows a Near Eastern Neolithic origin for domestic cattle and no indication of domestication of European aurochs. Proc Biol Sci 274:1377–1385 10.1098/rspb.2007.0020 PMC2176208 17412685

[21] Powell J, Talenti A, Fisch A, Hemmink JD, Paxton E, Toye P, Santos I, Ferreira BR, Connelley TK, Morrison LJ, Prendergast JGD (2023) Profiling the immune epigenome across global cattle breeds. Genome Biol 24:127 10.1186/s13059-023-02964-3 PMC10204299 37218021

[22] Utsunomiya YT, Milanesi M, Fortes MRS, Porto-Neto LR, Utsunomiya ATH, Silva M, Garcia JF, Ajmone-Marsan P (2019) Genomic clues of the evolutionary history of *Bos indicus* cattle. Anim Genet 50:557–568 10.1111/age.12836 31475748

[23] Regattieri E, Forti L, Drysdale RN, Mannella G, Hellstrom JC, Conati Barbaro C, Bonacossi DM, Zerboni A (2023) Neolithic hydroclimatic change and water resources exploitation in the Fertile Crescent. Sci Rep 13:45 10.1038/s41598-022-27166-y PMC9839760 36639410

[24] Bock RE, Kingston TG, de Vos AJ (1999) Effect of breed of cattle on transmission rate and innate resistance to infection with *Babesia bovis* and *B bigemina* transmitted by *Boophilus microplus*. Aust Vet J 77:461–464 10.1111/j.1751-0813.1999.tb12093.x 10451733

[25] Wambura PN, Gwakisa PS, Silayo RS, Rugaimukamu EA (1998) Breed-associated resistance to tick infestation in *Bos indicus* and their crosses with *Bos taurus*. Vet Parasitol 77:63–70 10.1016/s0304-4017(97)00229-x 9652384

[26] Pena MT, Miller JE, Wyatt W, Kearney MT (2000) Differences in susceptibility to gastrointestinal nematode infection between Angus and Brangus cattle in south Louisiana. Vet Parasitol 89:51–61 10.1016/s0304-4017(99)00229-0 10729645

[27] Démoulins T, Cherbuin JDR, Yimthin T, Eggerschwiler L, Adnan F, Jores J (2025) Beware of host immune responses towards bacteriophages potentially impacting phage therapy. Vet Res 56:170 10.1186/s13567-025-01600-1 PMC12357382 40817079

[28] Démoulins T, Yimthin T, Lindtke D, Eggerschwiler L, Siegenthaler R, Labroussaa F, Jores J (2024) Temperature impacts the bovine ex vivo immune response towards *Mycoplasmopsis bovis*. Vet Res 55:18 10.1186/s13567-024-01272-3 PMC10863263 38351086

[29] Démoulins T, Yimthin T, Zhang J, Leonard AS, Eggerschwiler L, Siegenthaler R, Kuhnert P, Gross JJ, Pausch H, Jores J (2026) Immune responses of *Bos indicus* versus *Bos taurus* cattle towards bluetongue virus or Schmallenberg virus differ significantly. Commun Biol 9:582 10.1038/s42003-026-09804-7 PMC13121734 41807544

[30] Yimthin T, Bourgeon M, Zhang J, Eggerschwiler L, Siegenthaler R, Schieck E, Torres-Puig S, Ruggli N, Demoulins T, Jores J (2025) Capsular polysaccharide of *Mycoplasma mycoides* subsp. *capri* contributes to phenotypic diversity promoting distinctive immune responses. PLoS Pathog 21:e1013386 10.1371/journal.ppat.1013386 PMC12716728 41385597

[31] Kuhnert P, Brodard I, Alsaaod M, Steiner A, Stoffel MH, Jores J (2020) *Treponema phagedenis* (ex Noguchi 1912) Brumpt 1922 sp. nov., nom. rev., isolated from bovine digital dermatitis. Int J Syst Evol Microbiol 70:2115–2123 10.1099/ijsem.0.004027 31999237

[32] Tassera L, Brodard I, Jores J, Kuhnert P (2026) Detection of *Treponema* species associated with bovine digital dermatitis in cattle using novel real-time PCR assays. J Vet Diagn Invest 38:451–457 10.1177/10406387261424230 PMC12962984 41782290

[33] Kuhnert P, Cippa V, Hardi-Landerer MC, Schmicke M, Ulbrich S, Locher I, Steiner A, Jores J (2019) Early infection dynamics of *Dichelobacter nodosus* during an ovine experimental footrot in contact infection. Schweiz Arch Tierheilkd 161:465–472 10.17236/sat00215 31298215

[34] Ruby J, Martin M, Passineau MJ, Godovikova V, Fenno JC, Wu H (2018) Activation of the innate immune system by treponema denticola periplasmic flagella through toll-like receptor 2. Infect Immun 86:10 10.1128/IAI.00573-17 PMC5736820 29084899

[35] Cheli R, Mortellaro C (1974) Digital dermatitis in cattle. Proceedings of the 8^th^ International Conference on Diseases of Cattle, p 208–213

[36] Evans NJ, Murray RD, Carter SD (2016) Bovine digital dermatitis: current concepts from laboratory to farm. Vet J 211:3–13 10.1016/j.tvjl.2015.10.028 27061657

[37] Staton GJ, Clegg SR, Ainsworth S, Armstrong S, Carter SD, Radford AD, Darby A, Wastling J, Hall N, Evans NJ (2021) Dissecting the molecular diversity and commonality of bovine and human treponemes identifies key survival and adhesion mechanisms. PLoS Pathog 17:e1009464 10.1371/journal.ppat.1009464 PMC8049484 33780514

[38] Moreira TF, Facury Filho EJ, Carvalho AU, Strube ML, Nielsen MW, Klitgaard K, Jensen TK (2018) Pathology and bacteria related to digital dermatitis in dairy cattle in all year round grazing system in Brazil. PLoS One 13:e0193870 10.1371/journal.pone.0193870 PMC5841792 29513739

[39] Pyakurel S, Caddey BJ, Dias AP, De Buck J, Morck DW, Orsel K (2025) Profiling bacterial communities in feedlot cattle affected with bovine foot rot and bovine digital dermatitis lesions using 16S rRNA gene sequencing and quantitative real-time PCR. BMC Microbiol 25:158 10.1186/s12866-025-03869-w PMC11924851 40114065

[40] Borregaard N (2010) Neutrophils, from marrow to microbes. Immunity 33:657–670 10.1016/j.immuni.2010.11.011 21094463

[41] Lahiri P, Young D, Penner R, Lesack K, Dias AP, Hannawayya R, De Buck J, Orsel K, Hollenberg MD, Johnson F, Golub L, Scaduto J, Dufour A, Cobo ER (2025) Proteolytic dysfunction in bovine digital dermatitis caused by *Treponema* spp. and the therapeutic use of non-antimicrobial matrix metalloproteinase inhibitors. Commun Biol 8:1503 10.1038/s42003-025-09011-w PMC12572248 41162547

[42] Lahiri P, Arrazuria R, Tan YL, De Buck J, Hollenberg MD, Orsel K, Cobo ER (2024) Proinflammatory CD14(high)CD16(low) monocytes/macrophages prevail in *Treponema phagedenis*-associated bovine digital dermatitis. Infect Immun 92:e0034223 10.1128/iai.00342-23 PMC10863414 38189287

[43] Evans NJ, Brown JM, Scholey R, Murray RD, Birtles RJ, Hart CA, Carter SD (2014) Differential inflammatory responses of bovine foot skin fibroblasts and keratinocytes to digital dermatitis treponemes. Vet Immunol Immunopathol 161:12–20 10.1016/j.vetimm.2014.05.005 25022220

[44] Jian W, Duangjinda M, Vajrabukka C, Katawatin S (2014) Differences of skin morphology in *Bos indicus*, *Bos taurus*, and their crossbreds. Int J Biometeorol 58:1087–1094 10.1007/s00484-013-0700-9 23824223

[45] Ahlen L, Holmoy IH, Nodtvedt A, Sogstad AM, Fjeldaas T (2022) A case-control study regarding factors associated with digital dermatitis in Norwegian dairy herds. Acta Vet Scand 64:19 10.1186/s13028-022-00635-0 PMC9375421 35964105

[46] Holzhauer M, Hardenberg C, Bartels CJ, Frankena K (2006) Herd- and cow-level prevalence of digital dermatitis in the Netherlands and associated risk factors. J Dairy Sci 89:580–588 10.3168/jds.S0022-0302(06)72121-X 16428627

[47] Lee H, Jeong S, Shin EC (2022) Significance of bystander T cell activation in microbial infection. Nat Immunol 23:13–22 10.1038/s41590-021-00985-3 34354279

[48] Lertmemongkolchai G, Cai G, Hunter CA, Bancroft GJ (2001) Bystander activation of CD8^+^ T cells contributes to the rapid production of IFN-gamma in response to bacterial pathogens. J Immunol 166:1097–1105 10.4049/jimmunol.166.2.1097 11145690

[49] Wilson-Welder JH, Nally JE, Alt DP, Humphrey SB, Olsen SC (2018) Short communication: lymphocyte proliferative responses in cattle naturally infected with digital dermatitis consist of CD8^+^ and gammadelta-T cells but lack CD4^+^ T cells. J Dairy Sci 101:8301–8307 10.3168/jds.2017-13913 29908808

[50] Eming SA, Koch M, Krieger A, Brachvogel B, Kreft S, Bruckner-Tuderman L, Krieg T, Shannon JD, Fox JW (2010) Differential proteomic analysis distinguishes tissue repair biomarker signatures in wound exudates obtained from normal healing and chronic wounds. J Proteome Res 9:4758–4766 10.1021/pr100456d 20666496

[51] Yager DR, Zhang LY, Liang HX, Diegelmann RF, Cohen IK (1996) Wound fluids from human pressure ulcers contain elevated matrix metalloproteinase levels and activity compared to surgical wound fluids. J Invest Dermatol 107:743–748 10.1111/1523-1747.ep12365637 8875960

[52] Springall R, Amezcua-Guerra LM, Gonzalez-Pacheco H, Furuzawa-Carballeda J, Gomez-Garcia L, Marquez-Velasco R, Mejia-Dominguez AM, Cossio-Aranda J, Martinez-Sanchez C, Bojalil R (2013) Interferon-gamma increases the ratio of matrix metalloproteinase-9/tissue inhibitor of metalloproteinase-1 in peripheral monocytes from patients with coronary artery disease. PLoS One 8:e72291 10.1371/journal.pone.0072291 PMC3741271 23951304

[53] Wolosowicz M, Prokopiuk S, Kaminski TW (2025) Matrix metalloproteinase-9 (MMP-9) as a therapeutic target: insights into molecular pathways and clinical applications. Pharmaceutics 17:1425 10.3390/pharmaceutics17111425 PMC12655286 41304763

[54] Bradley LM, Douglass MF, Chatterjee D, Akira S, Baaten BJ (2012) Matrix metalloprotease 9 mediates neutrophil migration into the airways in response to influenza virus-induced toll-like receptor signaling. PLoS Pathog 8:e1002641 10.1371/journal.ppat.1002641 PMC3320598 22496659

[55] Vikman S, Larsson A, Thulin M, Karlsson T (2023) Increased levels of a subset of angiogenesis-related plasma proteins in essential thrombocythemia. Ups J Med Sci 128:10 10.48101/ujms.v128.9194 PMC10084492 37051288

