# Supplementary material for: Bos indicus cattle resistant to digital dermatitis exhibit a balanced proinflammatory cytokine response upon exposure to Treponema phagedenis in contrast to Bos taurus cattle
Source: Vet Res. 2026 Oct 7;57:177. doi: 10.1186/s13567-026-01848-1 (PMC13644106; doi:10.1186/s13567-026-01848-1)
Supplement: Supplementary file 1 [file 13567_2026_1848_MOESM1_ESM.pdf]

**Animal**	**Location**	**Subspecies**	**Sex**	**Date of birth**	**Age**	**Previous infection***
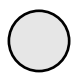	Posieux, FR	*Bos taurus*	F	2022-02-07	3 years and 8 months	No
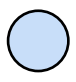	Posieux, FR	*Bos taurus*	F	2021-01-19	4 years and 8 months	No
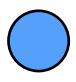	Posieux, FR	*Bos taurus*	F	2021-10-18	3 years and 11 months	No
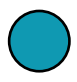	Posieux, FR	*Bos taurus*	F	2022-01-13	3 years and 8 months	No
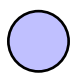	Posieux, FR	*Bos taurus*	F	2020-12-03	4 years and 10 months	No
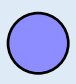	Posieux, FR	*Bos taurus*	F	2021-10-25	3 years and 11 months	**Yes**
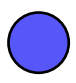	Posieux, FR	*Bos taurus*	F	2020-01-25	5 years and 8 months	No
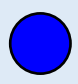	Posieux, FR	*Bos taurus*	F	26.09.2020	5 years	**Yes**
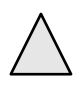	Bätterkinden, BE	*Bos indicus*	M	2025-06-09	5 months	No
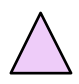	Bätterkinden, BE	*Bos indicus*	F	2023-07-25	2 years and 3 months	No
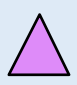	Bätterkinden, BE	*Bos indicus*	F	2008-01-24	17 years and 10 months	**Yes**
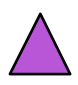	Bätterkinden, BE	*Bos indicus*	F	2019-11-16	6 years	No
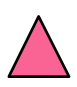	Bätterkinden, BE	*Bos indicus*	F	2024-12-20	10 months	No
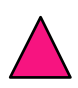	Bätterkinden, BE	*Bos indicus*	F	2023-08-29	2 years and 2 months	No
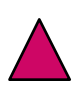	Bätterkinden, BE	*Bos indicus*	F	2024-04-01	1 year and 7 months	No
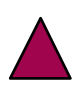	Bätterkinden, BE	*Bos indicus*	M	2024-11-10	1 year	No
