# Supplementary material for: Bos indicus cattle resistant to digital dermatitis exhibit a balanced proinflammatory cytokine response upon exposure to Treponema phagedenis in contrast to Bos taurus cattle
Source: Vet Res. 2026 Oct 7;57:177. doi: 10.1186/s13567-026-01848-1 (PMC13644106; doi:10.1186/s13567-026-01848-1)
Supplement: Supplementary file 2 [file 13567_2026_1848_MOESM2_ESM.pdf]

| Antibody | Source | Identifier |
|---|---|---|
| Anti-CD14 (Clone CAM36A; mouse Ig1; 1:800 dilution) | Kingfisher Biotech | Cat# WS0565B-100 |
| Anti-CD25 (Clone LCTB2A; mouse IgG3; 1:200 dilution) | Kingfisher Biotech | Cat# WS0597B-100 |
| Anti-CD11c (Clone BAQ153A; mouse IgM; 1:40 dilution) | Kingfisher Biotech | Cat# WS0519B-100 |
| Anti-CD16 (Clone KD1; mouse, IgG2a; 1:100 dilution) | Bio-Rad | Cat# MCA5665GA |
| Anti-CD172a (Clone CC149; mouse IgG2b; 1:200 dilution) | Bio-Rad | Cat# MCA2041GA |
| Anti-CD62L (Clone CC32; mouse IgG1; 1:320 dilution) | Bio-Rad | Cat# MCA1649G |
| Anti-WC1 (Clone CC15; mouse IgG2a; 1:800 dilution) | Bio-Rad | Cat# MCA838GA |
| Anti-CD21 (Clone CC51; mouse IgG2b; 1:800 dilution) | Bio-Rad | Cat# MCA5953GA |
| Anti-CD4-AF700 (Clone CC8; mouse IgG2a; 1:100 dilution) | Bio-Rad | Cat# MCA1653A700 |
| Anti-CD13 (Clone CC81; mouse IgG1; 1:100 dilution) | Bio-Rad | Cat# MCA2338GA |
| Anti-MHC-II-PE (Clone IL-A21; mouse IgG2a; 1:40 dilution) | Bio-Rad | Cat# MCA2445PE |
| Anti-CD3 (Clone MM1A; mouse IgG1; 1:200 dilution) | Bio-Rad | Cat# MCA6080 |
| Anti-CD8-Pacific Blue (Clone CC63; mouse IgG2a; 1:20 dilution) | Bio-Rad | Cat# MCA837PB |
| Anti-CD45RO-PE (clone IL-A116; mouse IgG3; 1:20 dilution) | Bio-Rad | Cat# MCA2434PE |
| Anti-CD335 (Clone AKS1; mouse IgG1; 1:100 dilution) | Bio-Rad | Cat# MCA2365GA |
| Anti-TNF-α-AF488 (Clone CC327; mouse IgG2b; 1:80 dilution) | Bio-Rad | Cat# MCA2334A488 |
| Anti-IFN-γ-A647 (Clone CC302; mouse IgG1; 1:160 dilution) | Bio-Rad | Cat# MCA1783A647 |
| Anti-IL-4-PE (Clone CC303; mouse IgG2a; 1:20 dilution) | Bio-Rad | Cat# MCA1820PE |
| Anti-IL-4-FITC (Clone CC303; mouse IgG2a; 1:20 dilution) | Bio-Rad | Cat# MCA1820F |
| Anti-CD20 (Clone MEM-97; mouse IgG1; 1:100 dilution) | TermoFisher | Cat# MA1-19008 |
| Anti-CCR7-Super Bright 645 (Clone 3D12; rat IgG2a; 1:80 dilution) | TermoFisher | Cat# 64-1979-42 |
| Anti-IL-17A-APC-ef780 (Clone Ebio64DEC17; mouse IgG1; 1:160 dilution) | TermoFisher | Cat# 47-7179-42 |
| Anti-FOXP3-Brilliant Violet 711 (Clone FJK-16S; rat IgG2a; 1:100 dilution) | TermoFisher | Cat# 407-5773-82 |
| Anti-TGF-β- DyLight 405 (Clone 1D11.16.8; mouse IgG1; 1:100 dilution) | Novus Biologicals | Cat# NBP3-08710V |
| Anti-mouse IgG2a-Super Bright 436 (Clone m2a-15F8; rat IgG1; 1:20 dilution) | TermoFisher | Cat# 62-4210-82 |
| Anti-mouse IgG2a-Super Bright 780 (Clone m2a-15F8; rat IgG1; 1:40 dilution) | TermoFisher | Cat# 78-4210-82 |
| Anti-mouse IgG3-PE-Cy5 (Goat IgG; 1:200 dilution) | SouthernBiotech | Cat# 1100-13 |
| Anti-mouse IgG2b-APC-Cy7 (Goat IgG; 1:400 dilution) | SouthernBiotech | Cat# 1090-19 |
| Anti-mouse IgG2b-PE-Cy7 (Goat IgG; 1:250 dilution) | SouthernBiotech | Cat# 1090-17 |
| Anti-mouse IgG1-PE-Cy7 (Clone RMG1-1; rat IgG; 1:640 dilution) | BioLegend | Cat# 406614 |
| Anti-mouse IgG1-Brilliant Violet 421 (Clone RMG1-1; rat IgG; 1:80 dilution) | BioLegend | Cat# 406616 |
| Anti-mouse IgM-BV786 (Clone R6-60.2; rat IgG2a; 1:100 dilution) | BD Bioscience | Cat# 564028 |
| **Others** | **Source** | **Identifier** |
| Zenon™ Mouse IgG1 Labeling Kit, AF647 | TermoFisher | Cat# Z25008 |
| Zenon™ Mouse IgG1 Labeling Kit, AF568 | TermoFisher | Cat# Z25006 |
| Zenon™ Mouse IgG1 Labeling Kit, AF700 | TermoFisher | Cat# Z25011 |
| LIVE/DEAD™ Fixable Yellow Dead Cell Stain Kit, for 405 nm | TermoFisher | Cat# L34968 |
| Brefeldin A Solution (1000X) | TermoFisher | Cat# 00-4506-51 |
